# Links Between Child Executive Function and Adjustment: A Three‐Site Study

**DOI:** 10.1111/cdev.14264

**Published:** 2025-05-29

**Authors:** Laure Lu Chen, Jean Anne Heng, Chengyi Xu, Michelle R. Ellefson, Miryam Edwards, Hana D'Souza, Elian Fink, Mikeda Jess, Louise Gray, Caoimhe Dempsey, Mishika Mehrotra, Siu Ching Wong, Catherine Wu, Brittany Huang, Jiayin Zheng, Zhen Wu, Rory T. Devine, Claire Hughes

**Affiliations:** ^1^ Centre for Child, Adolescent and Family Research University of Cambridge Cambridge UK; ^2^ Department of Psychological and Cognitive Sciences Tsinghua University Beijing China; ^3^ Faculty of Education University of Cambridge Cambridge UK; ^4^ Centre for Developmental Science University of Birmingham Birmingham UK; ^5^ School of Psychology Cardiff University Cardiff UK; ^6^ School of Psychology University of Sussex Brighton UK

**Keywords:** adjustment problems, cross‐cultural comparison, executive function, online home‐based assessment

## Abstract

Cross‐site comparisons indicate that East Asian children typically excel on tests of executive function (EF), but interpreting this contrast is made difficult by both the heavy reliance on testing in school settings and by the scarcity of studies that assess across‐site measurement invariance. Addressing these gaps, our study included remote home‐based assessments of EF for 1002 children (*M*_age_ = 5.19 years, SD = 0.51; 49% male) from England, Hong Kong, and mainland China, as well as parental ratings of externalizing and internalizing adjustment problems (data collected between June 2021 and December 2022). The models established partial scalar invariance but did not show clear site differences. Supporting the universal importance of EF for behavioral self‐regulation, EF task performance and parent‐rated externalizing problems showed similar inverse associations across sites.

## 1 Introduction

Executive function (EF) encompasses higher‐order cognitive processes such as planning, working memory, and impulse control, which are crucial for adaptive behavior. Thus, deficits in these component processes may serve as a transdiagnostic risk marker for diverse psychopathology outcomes. Supporting this view, strong EF has been reported to reduce the impact of genetic risk for developmental disorders (e.g., Johnson 2012) and interventions to boost EF capacity appear to ameliorate social and behavioral adjustment in middle childhood (Vaidya et al. 2020) and in the preschool years (Muir et al. 2023). However, researchers have recently proposed that the development of EF and its component processes should be viewed as closely entwined with specific task goals and sociocultural contexts (Doebel 2020). As such, there is a need to expand the cultural scope of investigations into relations between EF and adjustment.

Indeed, our current understanding of relations between EF and adjustment is largely based on findings from minority world samples (Yang et al. 2022), and is also limited by (i) the tendency for EF researchers to examine associations with either externalizing or internalizing problems, rather than considering these two dimensions of adjustment in tandem (Wang and Liu 2021); (ii) the heavy reliance on school settings for assessing child EF, which limits our ability to test the generalizability of results across testing contexts; and (iii) the scarcity of cross‐cultural studies that test measurement invariance to assess the across‐site conceptual equivalence of behavioral EF measures and parental ratings of child adjustment.

### 1.1 Do Links Between Executive Function and Adjustment Hold Across Cultural Contexts?

Externalizing difficulties (e.g., hyperactivity, conduct problems) and internalizing problems of anxiety and depression show significant co‐occurrence (Nivard et al. 2017). Evidence suggests that each of these dimensions is related to poor EF. For example, a meta‐analysis of 167 longitudinal studies (1098 effect sizes; *n* = 66,119 children below 18) showed weak but consistent associations between individual differences in EF and later externalizing problems (combined effect size of −0.11) and internalizing problems (combined effect size of −0.07) – the majority of which focused on Western, educated, industrialized, rich, democratic (WEIRD) samples (Yang et al. 2022). The paucity of child adjustment data for the Chinese samples limits conclusions regarding the universality of these EF‐adjustment associations.

Highlighting the danger of extrapolating results across different world regions, cross‐cultural studies demonstrate a puzzling methodological contrast. Specifically, studies using behavioral measures of EF indicate that East Asian children outperform their North American and European counterparts (for a systematic review, see Schirmbeck et al. 2020). Yet, survey studies indicate a contrast in the opposite direction, with Chinese parents reporting particularly low levels of inhibitory control (Grabell et al. 2015). Divergent results from behavioral and survey measures can also be found within the same study. For example, a multimethod comparison of British and Hong Kong preschoolers showed that average parental ratings of child hyperactivity were higher in Hong Kong than in the United Kingdom, but actigraphy records showed a contrast in the opposite direction (Chan et al. 2022).

At first glance, these contrasts between behavioral measures and parental ratings suggest an “eye of the beholder” effect, with culturally‐specific norms providing different benchmarks for evaluating child behavior. Considered more deeply, however, these findings add weight to recent calls for an understanding of EF as “in the service of specific goals” (Doebel 2020). While EF is traditionally viewed as a set of stable traits, short‐term fluctuations in task performance suggest links to motivational factors; for example, cognitive resource allocation may increase following reward information, but decrease in the context of high‐arousal task‐irrelevant stimuli (Pessoa 2009). As a result, the contrasting site differences that emerge from parental ratings and (school‐based) behavioral measures may simply reflect motivational influences on children's self‐regulation.

Indeed, studies that include different rewards report that children's willingness to delay gratification does not develop in a vacuum, but instead appears to align with social norms. For example, Japanese preschoolers appear more likely to wait for a snack than a gift, while their American counterparts show the opposite pattern (Yanaoka et al. 2022). Likewise, adopting a “bird in the hand” strategy of choosing small but immediate rewards may be adaptive in unreliable or resource‐poor environments (e.g., Miller‐Cotto et al. 2022). Consistent with this view, children's performance on the classic (marshmallows) delay of gratification task appears susceptible to proximal cues as to whether waiting is a rational choice. For example, a lab‐based study in which, before the Marshmallow delay of gratification task, American preschoolers were given a small set of well‐used crayons to create a drawing. All children were instructed to wait for the researcher to return with a new set of art supplies, but only 50% were assigned to a “reliable” condition where this promise was fulfilled. The other 50% who were assigned to an “unreliable” condition in which the researcher returned without the promised supplies showed much shorter waiting times in the Marshmallow task (Kidd et al. 2013). Strengthening the importance of contextual effects, recent work indicates that this “bird in the hand” effect is much weaker when the “crayons + Marshmallow” paradigm is delivered within a school setting, in which adults are generally viewed as reliable and supportive (Moffett et al. 2020).

In sum, like other areas of developmental science (Zaadnoordijk and Cusack 2022), the narrow geographical scope of current research limits our understanding of the role of EF in child adjustment, but emerging findings from exceptional studies highlight the importance of adopting a contextually infused approach. Building on this argument, below we consider whether findings from home‐based assessments match those from previous school‐based cross‐cultural studies.

### 1.2 Do Home‐Based Executive Function Assessments Yield Similar Results to Previous School‐Based Cross‐Cultural Studies?

Recent years have seen renewed interest in the effects of behavioral setting, sparked by the growing number of studies of “situated cognition” (e.g., Robbins and Aydede 2009). In a review of this field that focused on self‐control, Kalis et al. (2024) argue that definitions of self‐control as “the ability to pursue goals in the face of conflicting motivations” can usefully be reframed as “the set of skills by which individuals modulate their relationship to their environment (or more specifically, their behavioral setting).” In contrast with the traditional individualistic focus on cognitive mechanisms such as “mental willpower” (e.g., Baumeister and Tierney 2012), this approach recognizes that physical and social environments also matter. This view is echoed in studies of “co‐regulation” within parent–child dyads (Lobo and Lunkenheimer 2020) and supported by evidence that child‐researcher rapport boosts children's EF task performance (Gidron et al. 2020). From this perspective it follows that between‐site differences in school climate (Lan et al. 2011) may affect how well children engage with EF tasks (Hughes 2023), and hence contribute to the site differences in EF task performance noted in previous cross‐cultural studies. For instance, in contrast with the inquiry‐based learning models adopted in British schools, which encourage child participation and teacher–student engagement, even very young children in Hong Kong are expected to regulate behavioral control and follow adults' instructions (Wang et al. 2016).

An opportunity to take cross‐cultural studies beyond the classroom came from the construction of online task batteries during the COVID‐19 pandemic that enabled remote assessments of children's cognitive abilities (Zaadnoordijk and Cusack 2022). For example, in a study of 93 preschoolers from the Midwestern United States, a remote EF task battery showed high reliability, as well as significant relations to standardized tests of academic achievement (Ahmed et al. 2022). Adopting a similar approach, our remote assessment protocol enabled us to administer three EF tasks to children within their home settings.

### 1.3 Do Behavioral and Survey Measures of Self‐Regulation Show Cross‐Cultural Measurement Invariance?

Although it is well recognized that group differences in EF task performance may not reflect genuine differences in the underlying competence, the scarcity of cross‐cultural studies that assess measurement invariance raises the problem of “comparing chopsticks with forks” (Chen 2008). A rare cross‐cultural study to address this gap showed partial scalar invariance for a battery of computerized EF tasks completed during whole‐class sessions by 1311 9‐ to 16‐year‐old children from mainland China, Hong Kong, and the United Kingdom (Xu et al. 2020). Consistent with previous findings (reviewed by Schirmbeck et al. 2020), children from mainland China and Hong Kong both outperformed their British counterparts. Notably, Hong Kong children also displayed superior EF task performance relative to their counterparts residing in mainland China. One aim of the current study was to extend the developmental scope of this work, by examining across‐site measurement invariance in EF task performance for a much younger sample of 4‐ to 6.5‐year‐old children. Note that relations between EF and adjustment may be especially crucial in this developmental period, as the early school years bring both challenges and opportunities for children to develop their self‐regulatory skills (McClelland et al. 2015).

Assessments of measurement invariance are also scarce in multi‐site studies of parental ratings of child adjustment. In one recent exception, conducted early in the COVID‐19 pandemic, Foley et al. (2023) established partial scalar invariance of the Strengths and Difficulties Questionnaire (SDQ) among parents of more than 2000 3‐ to 8‐year‐olds from six countries. The subsequent cross‐site comparisons (based on a five‐factor model of SDQ scores) showed that British parents reported higher levels of child hyperactivity and emotion problems than parents in the other five countries (including China). Notably, however, our assessments of measurement invariance in the current study applied a two‐factor model based on the two‐latent‐factor construct of externalizing problems (i.e., hyperactivity and conduct problem subscales combined) and internalizing problems (i.e., emotional and peer problems combined), as this approach is recommended for community samples (Goodman et al. 2010).

### 1.4 The Present Study

Addressing the gaps in the present literature, this study had three preregistered aims (https://osf.io/7rkfe/). The first of these was to assess the universality of relations between EF task performance and parent‐rated child adjustment, by including young school‐aged children from three societies with distinct sociocultural landscapes and contexts: England, Hong Kong, and mainland China. Our second aim was to examine whether home‐based assessments of child EF would yield similar results to those gathered in previous (almost exclusively school‐based) cross‐cultural studies. Reflecting the notable gaps in the literature, our analyses for these twin aims were exploratory in nature. Our third aim was to test for measurement invariance; this aim was largely confirmatory as we sought to assess the prediction (based on research discussed above) that behavioral measures of EF and parental ratings of adjustment would each show across‐site conceptual equivalence.

## 2 Method

### 2.1 Participants and Procedure

The data for this study were collected between June 2021 and December 2022. Initially, a total of 1075 children and their families were recruited from England, Hong Kong, and mainland China. Seventy‐three children were excluded from our final analyses due to having a clinically relevant diagnosis of developmental delay (*n* = 34) or falling outside the age range of 4–6.5 years (*n* = 39), resulting in a final sample of 1002 participants. The English subsample comprised 320 children (*M*_age_ = 5.28 years, SD = 0.35; 43% male), recruited through online channels (e.g., *Facebook*) and primary schools across the country. The Hong Kong subsample comprised 289 children (*M*_*a*ge_ = 4.95 years, SD = 0.36; 52% male), recruited through kindergartens from 16 of the 18 administrative districts. The mainland Chinese subsample comprised 393 children (*M*_age_ = 5.30 years, SD = 0.64; 52% male), recruited through kindergartens, online channels (e.g., *RedNote* [Xiaohongshu], a Chinese social media platform loosely comparable to a mix of *Instagram* and *Pinterest*), and the researchers' and participants' own social networks. The participating mainland Chinese families came from urban areas in 20 provinces, three autonomous regions, and four municipalities.

The English subsample was primarily White (66%), with 11% identifying as mixed/multiple ethnic groups, 5% as Asian British, 1% as Black British, and 17% undisclosed. The Hong Kong subsample (97%) and the mainland Chinese subsample (89%) were predominately Han Chinese. All three sites showed a similar level of perceived social standing, but there were minor differences in the numbers of boys and girls (Cramér's *V* = 0.09), as well as modest between‐site contrasts in child age (*η* ^2^ = 0.09) and parental education (Cramér's *V* = 0.28; see Table 1). Parents in England (85%) and mainland China (81%) were more likely to have a bachelor's degree or higher than parents in the Hong Kong site (56%). It is worth noting that levels of education in the participating parents did not reflect local trends in higher education. For instance, approximately 34% of residents aged over 16 in the United Kingdom (Office of National Statistics 2021), 27% of residents aged over 15 in Hong Kong (Hong Kong Education Bureau, n.d.), and 23% of residents aged over 15 in mainland China have postsecondary or above education (China Statistical Yearbook, 2023). Given the demographic differences across sites, these measures were included as covariates in our models.

**TABLE 1:** Participants' demographic information.

	Whole sample (*n* = 1002)	England (*n* = 320)	Hong Kong (*n* = 289)	Mainland China (*n* = 393)	Effect sizes for group differences
Child					
Age (M, SD)	5.19 (0.51)	5.28 (0.35)	4.95 (0.36)	5.30 (0.64)	*η* ^2^ = 0.09
Boys (%)	492 (49%)	137 (43%)	149 (52%)	206 (52%)	Cramér's *V* = 0.09
Girls (%)	510 (51%)	183 (57%)	140 (48%)	187 (48%)	
Parent					
Degree education (%)	75%	85%	56%	81%	Cramér's *V* = 0.28
Social ladder (M, SD)	6.20 (1.51)	6.48 (1.44)	5.98 (1.55)	5.98 (1.50)	*η* ^2^ = 0.02

*Note:* Degree education (%) = percentage of parents having a bachelor's degree or higher. Social ladder = parents' perceived standing on a 10‐rung social ladder.

All procedures received favorable reviews from the Research Ethics Committees at the University of Cambridge, University of Birmingham, and Tsinghua University. Parents gave informed consent prior to the data collection. Parent and child measures were prepared in English and translated into Chinese by a panel of five bilingual researchers, using a collaborative and iterative translation method to maintain conceptual equivalence (Douglas and Craig 2007). Parents completed an online questionnaire booklet to report on adjustment problems and key participant characteristics (child age and gender, parental education, etc.). Children from England, Hong Kong, and mainland China were individually tested in English, Cantonese, and Mandarin, respectively, during online home testing sessions that lasted up to 75 min. These were conducted by trained research assistants via *Zoom* for the English and Hong Kong subsamples and via *Tencent Meeting* for the mainland Chinese subsample, and all online sessions were video recorded onto encrypted storage for later scoring. An important prerequisite for participation was having desktops/laptops or tablets with keyboards at home, which means that the study children across all three sites were similarly familiar with online activities. Parents were given instructions on how to set up their environments (e.g., screen and sound) prior to the online sessions to avoid technical difficulties. Before each task, a trained researcher encouraged the child to ask questions if he or she had difficulty understanding the task and asked parents to sit silently behind their child to ensure that each child completed the task battery independently.

### 2.2 Measures

#### 2.2.1 Executive Function

We assessed children's EF using the Flanker task, the Head‐Toes‐Knees‐Shoulders task, and the Backward Animal Span task, delivered in the same order across sites. These well‐validated tasks have simple instructions and clear visual/verbal stimuli, which help to maintain engagement and minimize fatigue in remote home‐based assessments (Ahmed et al. 2022). Each test trial was scored such that successful demonstration of competency received a score of either 1 (for the Flanker task and the Backward Animal Span task) or 2 (for the Head‐Toes‐Knees‐Shoulders task) and failure received a score of 0.

##### 2.2.1.1 The Flanker Task

We adapted the Flanker task (Zelazo et al. 2013), which was programmed using Gorilla software (Gorilla.sc) in England and PsychoPy in Hong Kong and mainland China. On each trial, the screen showed a line of cartoon fish, and children were told to ignore the peripheral fish (or “flanking stimuli”) and to respond to the centrally presented fish (or “target stimulus”) by pressing one of two marked keys positioned on the left and right of a QWERTY keyboard to indicate, as quickly as possible, the direction of the middle cartoon fish. The three sites used a similar QWERTY‐based keyboard layout. Children received feedback for the six practice trials but not for the 36 test trials, of which 17 presented incongruent stimuli (i.e., the target fish faced in a different direction to the flanking fish), and 19 presented congruent stimuli (i.e., the target fish faced the same direction as the flanking fish). These test trials were presented in a pseudo‐randomized order across participants. Trials with reaction times below 250 ms were classified as anticipatory responses, occurring when a participant either responded too eagerly before the stimulus appeared in the current trial or failed to release the key after the previous trial (Davidson et al. 2006). These trials were recoded as incorrect attempts, and their associated accuracy scores were excluded from analyses. Following Davidson et al. (2006), trials with reaction times exceeding a maximum of 2500 ms were recoded as failed attempts, and their associated accuracy scores were excluded from analyses. Responses to incongruent trials were summed together to give a possible range of 0–17, and we applied this overall incongruent accuracy as the single indicator of inhibitory control under the one‐factor solution for latent EF.

##### 2.2.1.2 The Head‐Toes‐Knees‐Shoulders (HTKS) Task

The HTKS task provides an engaging way of measuring cognitive flexibility. We adopted the three‐part HTKS task that includes four to six practice trials with feedback and then 10 test trials with no feedback in each part. On each test trial, children were scored 0 for an incorrect response, 1 for a self‐corrected response, and 2 for a fully correct response (Ponitz et al. 2009). Testing in Part 1 and Part 2 was discontinued if the child failed to receive a score of 4 or higher. In Part 1, children were instructed to respond *counter* to a single pair of commands (i.e., touch their head when told to touch their toes, and vice versa). In Part 2, children were instructed to remember an additional pair of commands (i.e., touch their shoulders when told to touch their knees, and vice versa). In Part 3, the two pairs of commands were switched, such that children were instructed to do the opposite, touching their head when told to touch their knees, touching their shoulders when told to touch their toes, and vice versa. Total scores range from 0 to 60. In the current study, the sum scores across 10 test trials in each part were used as three indicators of cognitive flexibility under the one‐factor solution for latent EF. Across all sites, children were instructed to stand at a distance where the researcher was able to see the full range of children's movements on camera.

##### 2.2.1.3 The Backward Animal Span (BAS) Task

We adopted an amended version of the Backward Digit Span task (Wechsler 2018), replacing digits with animals to make the task more engaging for remote administration. This three‐part task includes two practice trials with feedback and six test trials with no feedback. A researcher read a seemingly random series of animals and presented the pictures of those animals to children, and children were instructed to repeat the sequence aloud in the reverse order. Test trials began with two different two‐animal sequences (i.e., dog, horse; snail, snake), and animal span increased by adding a single animal in two sequences in Part 2 (i.e., fish, wolf, owl; whale, bee, crab) and Part 3 (i.e., bear, ant, frog, rat; bull, duck, worm, shark). Pilot work indicated these animals were easily recognizable to children across the three sites. Testing was discontinued if the child gave two consecutive incorrect responses. Total scores range from 0 to 6. The sum scores across two test trials in each part were used as three indicators of working memory under the one‐factor solution for latent EF.

#### 2.2.2 Adjustment Problems

Across sites, parents completed the 25‐item Strengths and Difficulties Questionnaire (SDQ; Goodman 2001), which has been translated into 40 different languages and shows acceptable internal consistency (Cronbach's *α* = 0.66) and test–retest reliability (*r* = 0.71) in parents of different cultural groups (Achenbach et al. 2008), making it suitable for cross‐cultural investigations of child adjustment. Parents indicated how true (0 = *not true*, 1 = *somewhat true*, 2 = *very true*) each of the items was for their child. Scores on 10 items representing the hyperactivity and conduct problems subscales of the SDQ were summed to give a dimension of externalizing problems. Scores on 10 items representing the emotional and peer problem subscales were summed to give a dimension of internalizing problems. In line with prior work using the Chinese version of the SDQ (Cronbach's *α*s = 0.57–0.69; Wang et al. 2023), Cronbach's *α* values of the externalizing and internalizing problems ranged from 0.63 to 0.77 in this study. However, when testing across‐site measurement invariance of the SDQ, we dropped three items (lies, fights, steals) from the externalizing problems dimension. Thus, our index of externalizing problems encompasses the following items: temper tantrums, disobedience, restlessness, distractibility, planning difficulties, and poor task persistence. We also dropped four items (good friend, popularity, best with adults, somatic symptoms) from the internalizing problems dimension. Thus, our index of internalizing problems encompasses the following items: worries, sadness, nervousness, fears, solitary play, peer victimization. These two dimensions improved internal consistency (Cronbach's *α*s = 0.64–0.79) and were used to construct a good‐fitting two‐factor measurement model (for goodness‐of‐fit statistics, see Table S1). Table S2 presents a detailed selection of the SDQ items in modeling the adjustment problems latent factors.

#### 2.2.3 Verbal Ability

The study children from England and mainland China completed the English and Chinese versions (respectively) of the receptive vocabulary task from the Wechsler Preschool and Primary Scale of Intelligence (WPSSI‐IV; Wechsler 2018). On each trial, a researcher read aloud a word, and the child was asked to point to the matching picture from an array of four pictures. Children received 1 point for each correct trial, and testing was discontinued after four consecutive errors. The English sub‐sample completed up to 38 items, and the mainland Chinese subsample completed up to 31 items. The Hong Kong sub‐sample completed the Hong Kong Cantonese Receptive Vocabulary Test (HKCRVT; Lee et al. 2019). In this task, children were awarded 1 point if they pointed to a picture matching a word uttered by the researcher. The Hong Kong subsample completed up to 65 items. We standardized raw total scores within each site such that each child's verbal ability was measured against peers in his/her own group.

#### 2.2.4 Key Participant Characteristics

We included child characteristics (age and gender) and family socioeconomic status (SES) as covariates. To index family SES, parents were asked to report their highest level of educational attainment and to complete the Ladder of Subjective Social Status (Singh‐Manoux et al. 2003), on which a 10‐rung ladder denotes the range of lowest to highest levels of perceived educational qualification, employment, and income status.

## 3 Results

### 3.1 Analytic Strategy

We analyzed the data using a latent variable framework in M*plus* version 8 (Muthén and Muthén 2017). We used confirmatory factor analysis (CFA) to specify measurement models and multiple‐groups CFA to establish cross‐cultural measurement invariance of performance‐based EF tasks and parental ratings of adjustment problems prior to comparing latent means. Structural equation modeling (SEM) was then used to examine the universality across the three sites in relations between child EF and adjustment. Table S3 presents the means, standard deviations, ranges, and distribution coefficients for raw scores on each of the main study measures. We applied a robust maximum likelihood estimator in each model to account for non‐normal distributions of our indicators (see Figure S1 for histograms; Brown 2015). Given a small amount of unplanned missingness (ranging from 1% to 15%, see Table S3), a default full information approach was applied in M*plus* so that all available cases were included in the analyses (Muthén and Muthén 2017). Unlike listwise deletion or mean replacement, this does not assume normality or complete random missingness and so produces less biased parameters and standard errors (Enders 2001).

We evaluated model fit using four criteria: Root Mean Square Error of Approximation (RMSEA) < 0.08, Comparative Fit Index (CFI) > 0.90, Tucker‐Lewis Index (TLI) > 0.90, and Standardized Root Mean Residual (SRMR) < 0.10. Model parameters are considered invariant if there is a nonsignificant change in RMSEA of 0.015 and in CFI of 0.01 for nested model comparisons (Chen 2007). Moreover, across‐site equality constraints result in a nonsignificant decrease in model fit if the change in SRMR is less than 0.03 at the metric invariance level or 0.015 at the scalar invariance level (Chen 2007).

### 3.2 Partial Scalar Invariance of Online Home‐Based Executive Function Assessments

As noted earlier, our initial analyses used accuracy scores to generate one inhibitory control indicator, three cognitive flexibility indicators, and three working memory indicators. We began by testing the fit of a measurement model in which all indicators were loaded onto one single EF latent factor both overall and within each site (see Table 3). In the whole sample, this one‐factor solution yielded a good fit, RMSEA = 0.032, 90% CI [0.011–0.051], CFI = 0.992, TLI = 0.985, SRMR = 0.021. There were correlated residuals for two cognitive flexibility indicators (i.e., Parts 1 and 2 in the HTKS task) and three working memory indicators (i.e., two‐animal, three‐animal, and four‐animal sequences in the BAS task). The correlated residuals reflected task‐specific variance and were therefore added to the subsequent models. Table 2 displays zero‐order correlations between the EF latent factor and other main study measures.

**TABLE 2:** Correlations between key study variables in the whole sample.

	1	2	3	4	5	6	7
1. Executive function	—						
2. Externalizing problems	−0.18**	—					
3. Internalizing problems	−0.05	0.32**	—				
4. Child age	0.36**	−0.12**	−0.02	—			
5. Child gender	0.08*	−0.11**	0.04	0.01	—		
6. Parental education	0.18**	−0.19**	−0.02	0.07*	−0.01	—	
7. Perceived social standing	−0.07*	−0.16**	−0.07*	0.00	−0.03	−0.29**	—

*Note:* Correlations involving the executive function, internalizing problems, and externalizing problems latent factors were calculated using a robust maximum likelihood estimator in M*plus*.

* *p* < 0.05.

** *p* < 0.01.

The across‐site measurement invariance of the one‐factor structure for latent EF was examined by placing progressive restrictions on the model (Brown 2015). A comprehensive summary of comparing model fit indices is shown in Table 3. The *configural invariant* model, in which the same factor structure was simultaneously estimated across sites while allowing for differently estimated parameters, provided an excellent fit to the data, RMSEA = 0.022, 90% CI [0, 0.048], CFI = 0.996, TLI = 0.992, SRMR = 0.026. The next *metric invariant* model that constrained factor loadings to be equal across sites provided a good fit to the data, RMSEA = 0.029, 90% CI [0, 0.049], CFI = 0.991, TLI = 0.987, SRMR = 0.047. Given the nonsignificant change in model fit compared to the less parsimonious model, it can be inferred that remote home‐based assessments of EF had the same structure and meaning for child respondents of different cultural groups (Brown 2015). In the *scalar invariant* model, however, imposing additional constraints of all equal intercepts across groups resulted in a significant deterioration in model fit, ΔRMSEA = 0.026, ΔCFI = −0.035. As full measurement invariance in all steps is often not supported, it is common to proceed with tests of mean differences or relations between constructs using the partially invariant factor (Putnick and Bornstein 2016). Partial (metric, scalar, or strict) invariance modeling enables estimates for a subset of noninvariant parameters (loadings, intercepts, or error variances) to vary across groups while constraining estimates of invariant parameters to equality (e.g., Foley et al. 2023; Putnick and Bornstein 2016). Recent findings from simulation studies reveal that partial scalar invariance is sufficient to recover latent means and path coefficients even when up to 80% of parameters are noninvariant (Pokropek et al. 2019).

**TABLE 3:** Tests of measurement invariance of a one‐factor measurement model of executive function across sites.

	*χ* ^2^	df	RMSEA [90% CI]	CFI	TLI	SRMR	Model comparison	ΔCFI	ΔRMSEA	ΔSRMR
Measurement model by site										
Whole sample (*n* = 1002)	21.94	11	0.032 [0.011, 0.051]	0.992	0.985	0.021				
England (*n* = 320)	12.46	11	0.020 [0, 0.064]	0.997	0.994	0.027				
Hong Kong (*n* = 289)	9.93	11	0 [0, 0.057]	1.000	1.000	0.022				
Mainland China (*n* = 393)	15.81	11	0.033 [0, 0.067]	0.990	0.981	0.027				
Measurement invariance										
M1: configural invariance	38.36	33	0.022 [0, 0.048]	0.996	0.992	0.026				
M2: metric invariance	57.56	45	0.029 [0, 0.049]	0.991	0.987	0.047	M2 – M1	−0.005	0.007	0.021
M3: scalar invariance	114.90**	57	0.055 [0.040, 0.070]	0.956	0.952	0.064	M3 – M2	−0.035	0.026	0.017
M4: partial scalar invariance	65.30	49	0.032 [0, 0.050]	0.988	0.984	0.051	M4 – M2	−0.003	0.003	0.004

Abbreviations: 90% CI, 90% confidence interval for RMSEA; CFI, Comparative Fit Index; RMSEA, Root Mean Square Error of Approximation; SRMR, Standardized Root Mean Square Residual; TLI, Tucker–Lewis Index; ΔCFI, difference in the robust CFI; ΔRMSEA, difference in the robust RMSEA; ΔSRMR, difference in the robust SRMR.

** *p* < 0.01.

Based on a review of modification indices, we allowed four indicator intercepts (i.e., Parts 2 and 3 in the HTKS task, and two‐animal and three‐animal sequences in the BAS task) to be freely estimated and achieved *partial scalar invariance*, with the proposed criteria for ΔRMSEA, ΔCFI, and ΔSRMR being met. However, it should be noted that further tests of item response theory (IRT) found that the four indicators with unequal intercepts did exhibit differential item functioning (DIF) (for complete results, see Table S4), suggesting that these test items involve abilities beyond the underlying EF skills (Walker 2011) and should be interpreted with caution. In sum, the EF latent factor exhibited partial scalar invariance, with equal form, equal loadings, and partially equal intercepts in all three sites.

### 3.3 Group Contrasts in Executive Function Performance and Adjustment Ratings

**FIGURE 1:**
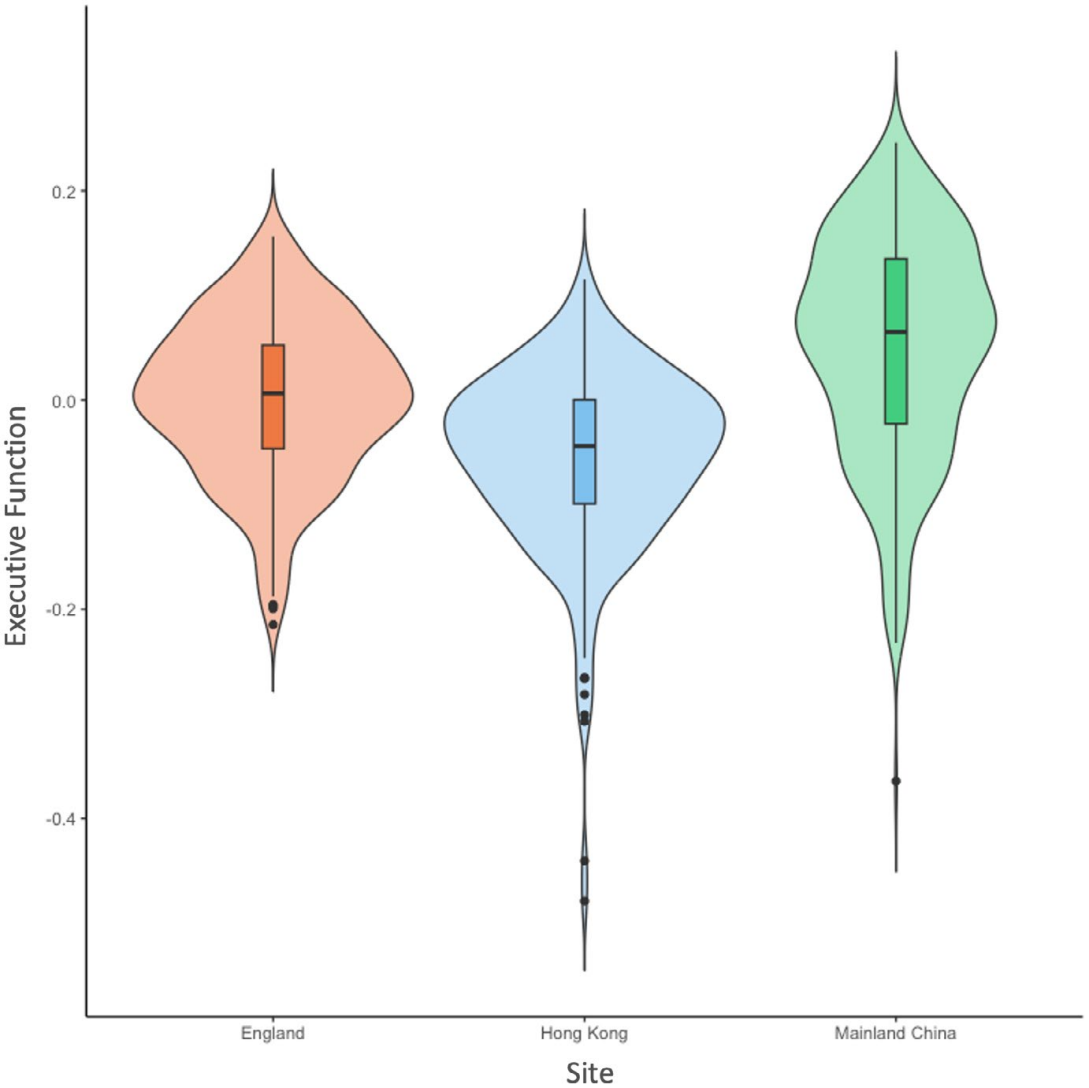
Violin plots showing latent means of executive function across sites after controlling for child and family covariates.

**FIGURE 2:**
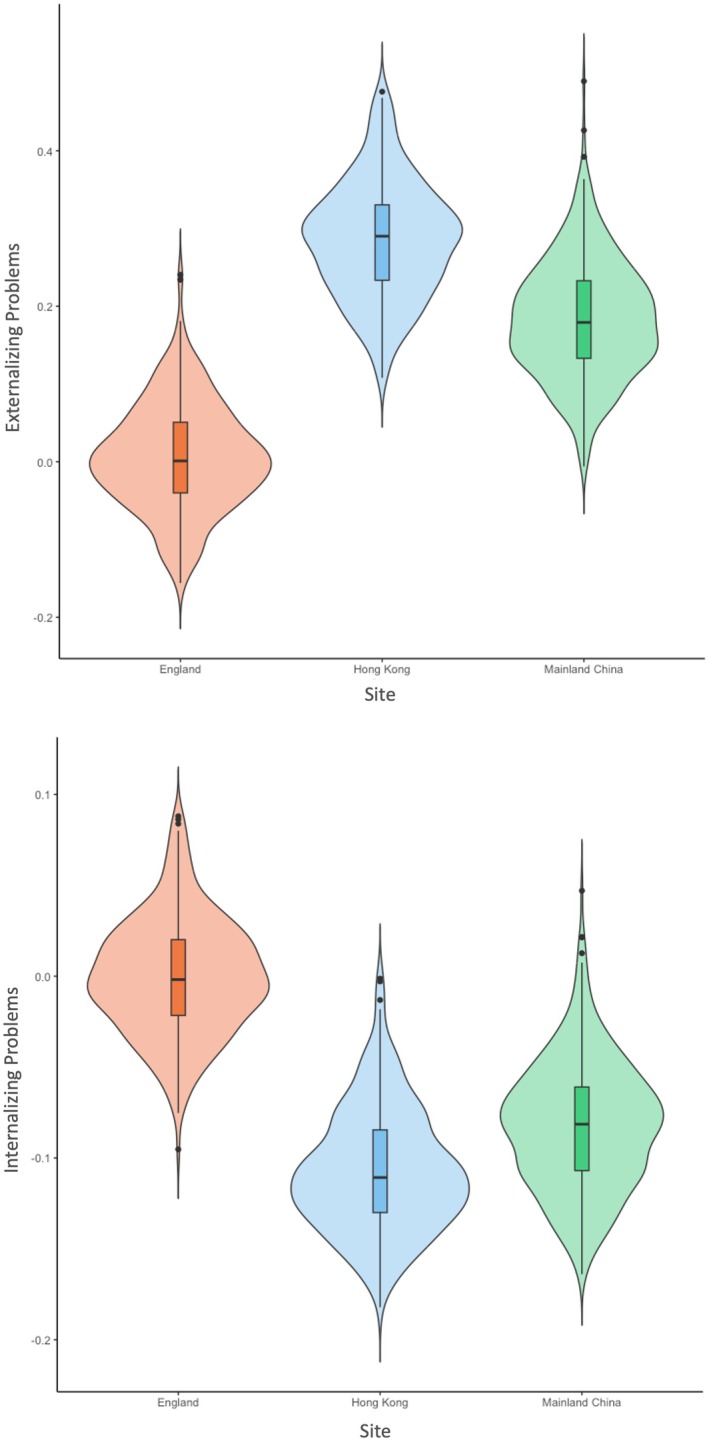
Violin plots showing latent means of adjustment problems across sites after controlling for child and family covariates.

Given that the partially scalar invariant modeling held, we evaluated the latent means of EF and adjusted total accuracy scores on each EF task across sites using analyses of covariance (ANCOVAs), with child age, gender, verbal ability, parental education, and perceived social standing treated as covariates (see Table 4). Interestingly, children from mainland China outperformed children from England and Hong Kong (see Figure 1). That said, the observed group differences were small in effect size, *η* ^2^ = 0.04. Examinations of adjusted total accuracy scores showed better performance on the cognitive flexibility task by mainland Chinese children than Hong Kong children, while both groups outperformed their English counterparts. For the working memory task, mainland Chinese and Hong Kong children obtained similar levels, and both outperformed their English counterparts. However, there was no significant difference in overall accuracy of the inhibitory control task across sites. Moreover, we employed the same stepwise procedures to establish partial scalar invariance of the two‐factor model of child adjustment, RMSEA = 0.051, 90% CI [0.043, 0.060], CFI = 0.932, TLI = 0.918, SRMR = 0.059 (for complete results, see Table S1), and IRT models suggested that five items exhibited DIF between English and Chinese child respondents, with no evidence of DIF between the Hong Kong and mainland Chinese subsamples (for complete results, see Table S5). The latent means of adjustment problems were also examined after adjusting for the same control covariates: Hong Kong children were rated highest in externalizing problems, while English children were rated highest in internalizing problems (see Figure 2).

**TABLE 4:** Mean comparisons of executive function task performance across Sites.

	England (*n* = 320)		Hong Kong (*n* = 289)		Mainland China (*n* = 393)		*F* ratio	*η* ^2^	Group comparison (post hoc tests)
	*M*	SE	*M*	SE	*M*	SE			
Executive function latent means	−0.02	0.01	−0.02	0.01	0.05	0.01	17.73 (*p* < 0.001)	0.04	MC > ENG ≈ HK
Adjusted total accuracy scores on each task									
Inhibitory control (Flanker)	9.81	0.31	9.72	0.36	9.81	0.26	0.02 (*p =* 0.977)	0	ENG ≈ MC ≈ HK
Cognitive flexibility (HTKS)	34.73	0.81	39.75	0.93	43.14	0.69	31.76 (*p* < 0.001)	0.07	MC > HK > ENG
Working memory (BAS)	2.64	0.08	3.03	0.09	3.00	0.07	7.87 (*p* < 0.001)	0.02	HK ≈ MC > ENG

*Note:* Results here were based on ANCOVAs, controlling for child and family covariates.

Abbreviations: ENG, England; HK, Hong Kong; MC, mainland China.

### 3.4 Culturally Universal Associations Between Executive Function and Adjustment

Building on the good‐fitting measurement invariance model across sites, we applied multiple‐groups SEM to examine cultural universality in associations between children's performance on behavioral assessments of EF and parental ratings of adjustment problems. Initially, we used the whole sample to regress latent factors for both internalizing and externalizing problems onto the EF latent factor, with child age, child gender, verbal ability, parental education, and perceived social standing treated as covariates. This model provided an acceptable fit to the data, RMSEA = 0.036, 90% CI [0.032, 0.040], CFI = 0.932, TLI = 0.918, SRMR = 0.041, which accounted for 50% of the variance in latent EF and 11% of the variance in latent externalizing problems, but did not significantly explain the variance in latent internalizing problems.

Next, we progressively placed equality constraints on parameters and path coefficients (for step‐by‐step model fit indices, see Table 5). Our multiple‐groups SEM met the condition of partial scalar invariance (i.e., equal form, equal factor loadings, and partially equal intercepts). This suggests that indicators have equivalent relations with the underlying construct in all three sites, enabling us to conduct meaningful across‐site comparisons of the direction and strength of paths between EF and adjustment problems. Even with all equality constraints on the regression paths, the model provided an acceptable fit to the data, RMSEA = 0.035, 90% CI [0.029, 0.040], CFI = 0.932, TLI = 0.925, SRMR = 0.061 (for unstandardized parameter estimates, see Figure S2). In addition, multiple‐groups comparisons revealed the difference between the partial scalar invariance measurement model (M1) and the model with all equality constraints (M5) was not statistically different (ΔCFI < 0.01), indicating that the paths linking EF with externalizing problems and internalizing problems were roughly universal for children of all three cultural groups. Our analyses showed that latent EF was inversely associated with externalizing problems, *B* = −0.45, SE = 0.13, *t* = −3.74, *p* < 0.001, but was unrelated to internalizing problems, *B* = 0.04, SE = 0.12, *t* = 0.30, *p* = 0.761.

**TABLE 5:** Structural relations between executive function and adjustment problems in the whole sample and across sites.

	*χ* ^2^	df	RMSEA [90% CI]	CFI	TLI	SRMR	Model comparison	ΔCFI
M0: Whole sample SEM	529.93***	241	0.036 [0.032, 0.040]	0.932	0.918	0.041		
Multiple‐groups SEM								
M1: equal factor loadings + partially equal intercepts	1029.39***	759	0.034 [0.029, 0.039]	0.937	0.928	0.057		
M2: M1 + equal path (ExT on EF)	1031.66***	761	0.034 [0.029, 0.039]	0.937	0.928	0.057	M2–M1	0
M3: M1 + equal path (InT on EF)	1031.35***	761	0.034 [0.029, 0.039]	0.937	0.928	0.057	M3–M1	0
M4: M1 + equal paths (ExT on EF and InT on EF)	1034.78***	763	0.034 [0.029, 0.039]	0.937	0.928	0.057	M4–M1	0
M5: M1 + all paths equal	1084.56***	793	0.036 [0.030, 0.040]	0.932	0.925	0.061	M5–M1	−0.005

Abbreviations: EF, executive function; ExT, externalizing problems; InT, internalizing problems; M0, unconstrained model; M1, constrained model in which all factor loadings and specific intercepts were constrained; M2, constrained model in which all factor loadings, specific intercepts, and the path coefficient between EF and externalizing problems were constrained; M3, constrained model in which all factor loadings, specific intercepts, and the path coefficient between EF and internalizing problems were constrained; M4, constrained model in which all factor loadings, specific intercepts, and the path coefficients between EF and both dimensions of adjustment problems were constrained; M5, constrained model in which all factor loadings, specific intercepts, and all path coefficients were constrained.

*** *p* < 0.001.

**FIGURE 3:**
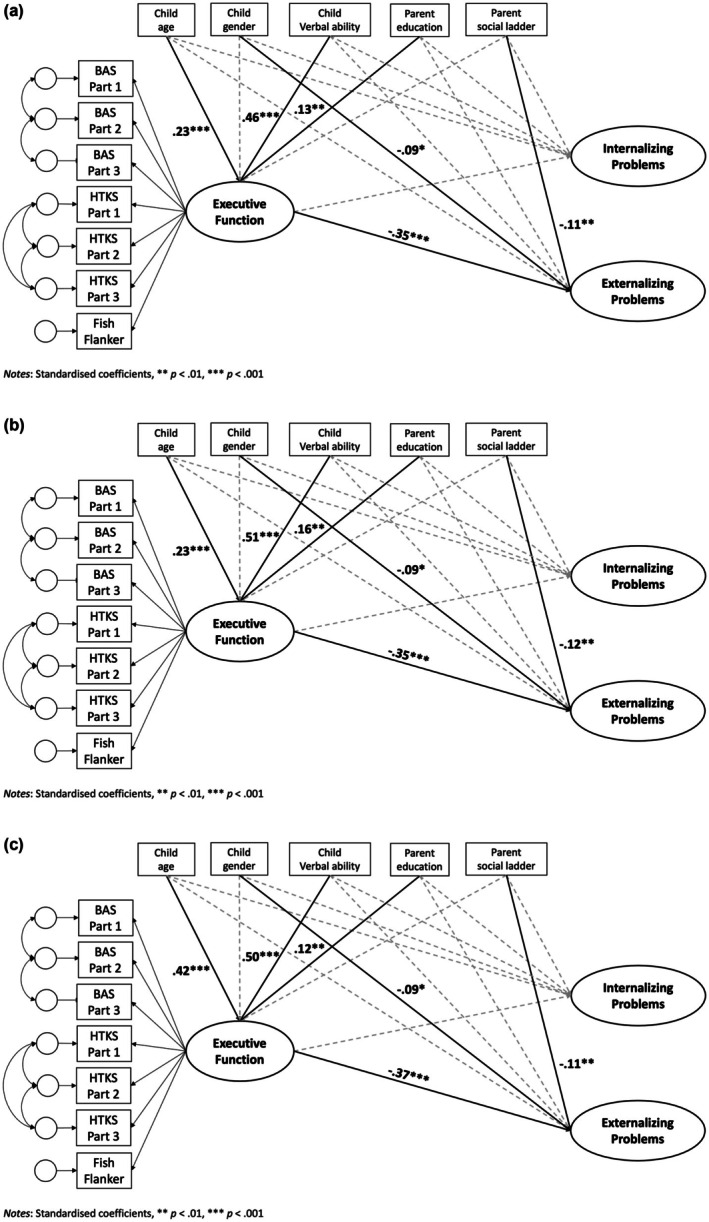
Standardized robust maximum likelihood estimates for paths between executive function and adjustment problems (with all equality constraints placed). (a) England. (b) Hong Kong. (c) Mainland China. Dash lines represent nonsignificant paths. Solid lines represent significant paths. **p < 0.01. ***p < 0.001.

As shown in Figure 3, standardized estimates within each site demonstrated similar associations between EF and externalizing problems: *β*_England_ = −0.35, SE = 0.09, *t* = −3.91, *p* < 0.001, *β*_Hong Kong_ = −0.35, SE = 0.09, *t* = −3.86, *p* < 0.001, and *β*_mainland China_ = −0.37, SE = 0.09, *t* = −4.03, *p* < 0.001. In short, across different cultural groups, a one‐unit increase in EF task performance yielded a 0.3–0.4 unit decrease in parent‐rated externalizing problems.

## 4 Discussion

This multisite study of 1002 preschool children living in England, Hong Kong, and mainland China yielded three sets of findings. First, consistent with previous meta‐analytic evidence from studies mostly involving WEIRD samples (e.g., Yang et al. 2022), EF scores showed a significant inverse association with externalizing problems, with similar magnitudes across sites (−0.37 ≤ *β* ≤ −0.35), even when child characteristics, verbal ability, and family SES were taken into account. Constraining path coefficients to equality across sites did not significantly reduce model fit, suggesting that this pathway equally applied to English, Hong Kong, and mainland Chinese children. By contrast, EF task performance was unrelated to parental ratings of internalizing problems, strengthening the view that this association only becomes significant later in life (e.g., Wang and Liu 2021). Second, challenging reports of superior EF in Hong Kong children made in previous school‐based studies (e.g., Xu et al. 2020), our remote assessments showed group differences that were modest in size but highlighted an advantage for mainland Chinese children relative to their counterparts in both England and Hong Kong. Third, via formal tests of measurement invariance, our partial scalar invariant models supported comparable relations between observed measures and the latent constructs of EF and adjustment across the three sites. Below, we address each finding in turn.

### 4.1 Executive Function Is Universally Associated With Externalizing Problems

Drawing upon data from the NICHD Study of Early Child Care and Youth Development (SECCYD), poor EF has been found to predict high levels of teacher‐rated externalizing and internalizing problems in primary school, independent of the co‐occurrence of both adjustment problems (Wang and Liu 2021). Extending to the preschool years, a meta‐analysis of 22 studies (*n* = 4021 preschoolers) conducted in the United States, Sweden, United Kingdom, Netherlands, Canada, Italy, and Austria has shown a medium effect size (*ESzr* = 0.22) for the correlation between overall EF impairments and externalizing problems (Schoemaker et al. 2013). However, few studies have investigated the association between early EF and internalizing problems. This is alarming, considering that children at age 4.5 years with greater levels of externalizing problems are more susceptible to internalizing adjustment difficulties throughout childhood and adolescence (Davis et al. 2015). The current study therefore contributes to the field by (i) moving beyond the WEIRD context through the inclusion of two East Asian sites; (ii) supporting the cultural universality of an inverse association between EF and externalizing problems; and (iii) demonstrating that this association was relatively specific, in that EF task performance was unrelated to parental ratings of 4‐ to 6.5‐year‐olds' internalizing problems.

### 4.2 Similarities and Differences in Executive Function Task Performance Across Cultural Contexts

Including children from both Hong Kong and mainland China enabled us to avoid perpetuating the stereotypes of East–West comparisons to consider within‐culture contrasts. Previous EF research indicates that school‐aged children and adolescents from Hong Kong outperform their counterparts from both the United Kingdom (Wang et al. 2016) and mainland China (Xu et al. 2020). Other studies that evaluated the psychometric properties of EF tasks among children from more than one East Asian society (e.g., Wanless et al. 2011) have rarely reported mean differences at the latent level. Thus, it is striking that, in the current study, kindergarteners from mainland China outperformed their counterparts in Hong Kong and England, who performed at roughly similar levels. Notably, our study is not the first to report a lack of group differences in EF between Hong Kong and European samples. A study of 170 8‐ to 10‐year‐olds from Hong Kong and Germany reported no significant contrast in inhibitory control, with this null result being attributed to the use of a Stroop task that taps more cognitive than behavioral inhibition (Schirmbeck et al. 2021). Along this line, our findings of site contrasts in the Heads‐Toes‐Knees‐Shoulders task that requires considerable behavioral self‐regulation (Ponitz et al. 2009) add weight to the view that group differences hinge upon behavioral control.

A key element of our home‐based (vs. school‐based) assessments is parental presence. While the participating parents were encouraged to be as quiet as possible during online testing sessions, with frequent reminders to not engage throughout the protocol, the potential influence of parental presence on children's task performance warrants careful consideration. For example, a meta‐analysis of 62 studies (*n* = 4405) indicated an overall adverse effect (*d* = −0.24) of the presence of an observer on cognitive task performance (Eastvold et al. 2012). Reassuringly, however, their results were moderated by cognitive domains, such that a third‐party presence was unrelated to EF task performance.

It is important to acknowledge that differences between our results and those from prior cross‐cultural studies may reflect between‐site contrasts in the impact of the COVID‐19 pandemic. Data collection in England took place in the spring and summer of 2021—several months after the last national lockdown and almost a year after the only major period of school closures. Data collection in Hong Kong and mainland China took place in the spring and summer of 2022—a period that encompassed multiple short‐term district‐specific lockdowns in mainland China and citywide lockdown in Hong Kong. Thus, pandemic‐related school disruption may have disproportionately affected the Hong Kong subsample. Additionally, pandemic‐related restrictions (e.g., stay‐at‐home policies) may have elevated levels of household chaos (Wang et al. 2021), which has been shown to exert direct and indirect effects—via parental responsiveness—on EF development (Andrews et al. 2021). While many of the study children in mainland China also lived in high‐density housing environments and experienced mass school closures, it is worth noting that Hong Kong families have only around half the per capita living space of families in urban mainland China, despite much higher average family incomes (Legislative Council 2022; Zhang 2023). As such, remote assessments of Hong Kong children were sometimes conducted in home settings that appeared crowded. From the perspective of Kalis et al.'s (2024) proposed “situated science of self‐control,” behavioral setting contrasts may well have contributed to site differences in EF task performance.

### 4.3 Online Home‐Based Assessments of Executive Function Show Across‐Site Measurement Invariance

Obtaining partial scalar invariance indicates the suitability of remote home‐based EF assessments across different cultural groups, strengthening findings from previous (school‐based) cross‐cultural studies in which EF was assessed individually (Schirmbeck et al. 2022) or in whole‐class sessions (Xu et al. 2020). Whole‐class testing offers a real advantage of efficiency but may not be feasible for younger children who need more one‐to‐one attention and support. Remote assessments dramatically reduce travel time and cost, enabling researchers to collaborate across regional and national borders and providing opportunities to test the generalizability of study findings (Kalis et al. 2024). The current study also increases confidence in the feasibility of remote assessments across tasks with three different response modalities (button press for the Flanker task, gross motor movement for the Head‐Toes‐Knees‐Shoulders task, and verbal responses for the Backward Animal Span task). This is reassuring as changes in response modality help maintain children's attention and engagement during online testing sessions. At the same time, however, the presence of noninvariant intercepts and DIF means that our results need to be treated with caution. For example, animal picture stimuli in the span task, although equally familiar across sites, only had the same number of syllables in Cantonese and Mandarin. Future cross‐cultural research should take into account stimulus‐length effects when devising assessment tools.

Consistent with previous examinations of the SDQ's psychometric properties, we found support for partial scalar invariance across the three sites. Note that there is a precedent for removing SDQ items; for example, partial scalar invariance was established after dropping five reverse‐worded items in a 7‐site self‐report study (Duinhof et al. 2020) and three items (exposure to bullying, fighting, stealing) in a 6‐site parent‐report study (Foley et al. 2023) In determining the removal of items, while Duinhof et al. (2020) noted effects of reversal ambiguity on item interpretations, Foley et al. (2023) considered the likely influence of socio‐historical contexts (such as mass school closures during the pandemic). When we specified the latent structure of adjustment problems, three items in the externalizing problems dimension and four items in the internalizing problems dimension were removed. The low factor loadings in our analyses suggest that prolonged school closures and social distancing measures may have made these seven items difficult for parents to answer. The presence of DIF in the five items with unequal thresholds between English and Chinese child respondents suggests between‐culture group differences in the underlying distributions of additional abilities (Walker 2011), underscoring the need for further investigations of measuring adjustment problems in a culturally appropriate manner.

### 4.4 Limitations and Future Directions

The use of online home‐based assessments is a distinctive feature of our study. More work is needed to assess the potential impact of assessment setting/modality (e.g., home vs. school settings, human‐to‐computer vs. human‐to‐human interactions) on other cognitive tests (Ahmed et al. 2022; Zaadnoordijk and Cusack 2022). Despite large‐scale data collection efforts, our subsamples in each site are not regionally representative (e.g., in terms of parental education levels). The participating children were primarily from urban areas, where they often have greater exposure to educational opportunities compared to those in rural settings. This underscores the need to replicate these findings in more diverse and underserved populations, particularly in majority world countries. From an intervention perspective, while our findings of significant associations between EF and adjustment support the view that improving EF may reduce symptoms of psychopathology (e.g., Vaidya et al. 2020), longitudinal research is needed to clarify directionality and elucidate potentially culture‐specific pathways of influence. Although measurement invariance is a prerequisite in cross‐cultural comparisons, researchers should be mindful of socio‐historical contexts influencing children's knowledge, beliefs, and values that may also affect children's performance on cognitive tests (Hughes 2023).

## 5 Conclusions

To our knowledge, this work represents the first multi‐site study to obtain direct assessments of EF using fully remote methods, while ensuring a standardized approach to the task battery across all English, Hong Kong, and mainland Chinese preschoolers. This is notable, given the twin challenges for researchers to adopt sustainable methodologies and to widen the geographical reach of their samples. Establishing partial scalar invariance permits this study to provide first evidence that associations between EF task performance and parental ratings of adjustment extend to the East Asian context. Our failure to replicate previously reported substantial East–West contrasts in EF highlights the need for future studies to include comprehensive task batteries delivered across multiple settings (e.g., at home and in school) to enable in‐depth analysis of sociocultural influences on EF.

## Author Contributions

**Laure Lu Chen:** conceptualization, data curation, formal analysis, original draft preparation, reviewing and editing. **Jean Anne Heng:** conceptualization, methodology, investigation, original draft preparation, reviewing and editing. **Chengyi Xu:** conceptualization, methodology, investigation, validation, original draft preparation, reviewing and editing, funding acquisition. **Michelle R. Ellefson:** methodology, reviewing and editing, supervision. Hana D'Souza: funding acquisition, project administration, supervision. Elian Fink: conceptualization, methodology, funding acquisition, project administration, supervision. Zhen Wu: funding acquisition, project administration, supervision. **Rory T. Devine:** conceptualization, methodology, reviewing and editing, project administration, supervision. **Claire Hughes:** conceptualization, methodology, original draft preparation, reviewing and editing, funding acquisition, project administration, supervision.

## Conflicts of Interest

The authors declare no conflicts of interest.

## Supporting information


Data S1.

## Data Availability

The data necessary to reproduce the analyses presented here are publicly accessible; data are available at the following URL: https://osf.io/7rkfe/. The analytic code necessary to reproduce the analyses presented here is publicly accessible; *Mplus* syntax is available at the following URL: https://osf.io/7rkfe/. The materials necessary to attempt to replicate the findings presented here are available from the corresponding author upon reasonable request. The analyses presented here were preregistered; the preregistration is available at the following URL: https://osf.io/7rkfe/.
