# Supplementary material for: Links Between Child Executive Function and Adjustment: A Three‐Site Study
Source: Child Dev. 2025 May 29;96(5):1590–604. doi: 10.1111/cdev.14264 (PMC12379854; doi:10.1111/cdev.14264)
Supplement: Supplementary file 1 — Data S1. [file CDEV-96-1590-s001.docx]

**Supplementary Materials**

Table of Contents

[Table S1](#_Toc175219541) [Tests of Measurement Invariance of a Two-Factor Measurement Model of Adjustment Problems across Sites 2](#_Toc175219542)

[Table S2](#_Toc175219543) [Selection of the SDQ Items for a Two-Factor Measurement Model of Adjustment Problems 3](#_Toc175219544)

[Table S3](#_Toc175219545) [Summary of Descriptive Statistics for Executive Function Tasks and the SDQ 4](#_Toc175219546)

[Table S4](#_Toc175219547) [Information Criteria of the Uniform MIMIC-DIF Testing Procedure for Four Items of the EF Latent Variable, Model Comparisons, and DIF Effects (MLR estimation) 5](#_Toc175219548)

[Table S5](#_Toc175219549) [Information Criteria of the Uniform MIMIC-DIF Testing Procedure for Nine Items of the Child Adjustment Latent Variables, Model Comparisons, and DIF Effects (MLR estimation) 6](#_Toc175219550)

[Figure S1](#_Toc175219551) [Histograms with Normal Curves for Executive Function Tasks 7](#_Toc175219552)

[Figure S2](#_Toc175219553) [Unstandardized Robust Maximum Likelihood Estimates for Paths between Executive Function and Adjustment Problems (With All Equality Constraints Placed) 13](#_Toc175219554)

[Mplus Codes 14](#_Toc175219555)

[1. Configural, Metric, and Scalar Invariance Models of Executive Function 14](#_Toc175219556)

[2. Partial Scalar Invariance Model of Executive Function 15](#_Toc175219557)

[3. Configural, Metric, and Scalar Invariance Models of Adjustment Problems 16](#_Toc175219558)

[4. Partial Metric Invariance Model of Adjustment Problems 17](#_Toc175219559)

[5. Partial Scalar Invariance Model of Adjustment Problems 19](#_Toc175219560)

[6. The Uniform DIF Testing (MLR Estimation) 22](#_Toc175219561)

[7. Multiple-Group Structural Equation Modeling without Constraints 23](#_Toc175219562)

[8. Multiple-Group Structural Equation Modeling with Constraints 26](#_Toc175219563)

# **Table S1**

# *Tests of Measurement Invariance of a Two-Factor Measurement Model of Adjustment Problems across Sites*

|  | *χ*^2^ | *df* | RMSEA  [90% CI] | CFI | TLI | SRMR | Model comparison | ΔCFI | ΔRMSEA | ΔSRMR |
| --- | --- | --- | --- | --- | --- | --- | --- | --- | --- | --- |
| ***Measurement model by site*** |  |  |  |  |  |  |  |  |  |  |
| Whole sample (*n* = 975) | 205.03*** | 58 | 0.051  [.044, .059] | 0.936 | 0.914 | 0.047 |  |  |  |  |
| England (*n* = 299) | 108.73*** | 58 | 0.054  [.038, .070] | 0.934 | 0.911 | 0.052 |  |  |  |  |
| Hong Kong (*n* = 287) | 113.61*** | 58 | 0.058  [.042, 0.073] | 0.930 | 0.906 | 0.063 |  |  |  |  |
| Mainland China (*n* = 389) | 109.24*** | 58 | 0.048  [0.034, .061] | 0.941 | 0.921 | 0.051 |  |  |  |  |
| ***Measurement invariance*** |  |  |  |  |  |  |  |  |  |  |
| M1: configural invariance | 331.54*** | 174 | 0.053  [.044, .061] | 0.935 | 0.913 | 0.055 |  |  |  |  |
| M2: metric invariance | 386.25*** | 196 | 0.055  [.047, .063] | 0.922 | 0.907 | 0.066 | M2 – M1 | -0.013 | 0.002 | 0.011 |
| M3: partial metric invariance | 344.19*** | 190 | 0.050  [.041, .058] | 0.937 | 0.922 | 0.058 | M3 – M1 | 0.002 | -0.003 | 0.003 |
| M4: partial scalar invariance | 360.52*** | 194 | 0.051  [.043, .060] | 0.932 | 0.918 | 0.059 | M4 – M3 | -0.005 | 0.001 | 0.001 |

*Note.* RMSEA, Root Mean Square Error of Approximation; 90% CI, 90% confidence interval for RMSEA; CFI, Comparative Fit Index; TLI, Tucker–Lewis Index; SRMR, Standardized Root Mean Square Residual. ΔCFI = difference in the robust CFI; ΔRMSEA = difference in the robust RMSEA; ΔSRMR = difference in the robust SRMR. *** *p* < .001.

# **Table S2**

# *Selection of the SDQ Items for a Two-Factor Measurement Model of Adjustment Problems*

| Item | Inclusion | Exclusion |
| --- | --- | --- |
| **F1: Externalizing problems** |  |  |
| *Conduct problems* |  |  |
| 5. Temper tantrums | √ |  |
| 7. Obedient* | √ |  |
| 12. Fights and bullies children |  | √ |
| 18. Lies and cheats |  | √ |
| 22. Steals |  | √ |
| *Hyperactivity* |  |  |
| 2. Restless | √ |  |
| 10. Constantly fidgeting | √ |  |
| 15. Easily distracted | √ |  |
| 21. Thinks things out* | √ |  |
| 25. Sees task through to end* | √ |  |
| **F2: Internalizing problems** |  |  |
| *Emotion problems* |  |  |
| 3. Complaints of headaches |  | √ |
| 8. Many worries | √ |  |
| 13. Often unhappy and tearful | √ |  |
| 16. Nervous and clingy | √ |  |
| 24. Many fears and easily scared | √ |  |
| *Peer problems* |  |  |
| 6. Solitary play | √ |  |
| 11. At least one good friend* |  | √ |
| 14. Liked by other children* |  | √ |
| 19. Picked on and bullied | √ |  |
| 23. Gets on better with adults |  | √ |

*Note*. *Reverse scored.

# **Table S3**

# *Summary of Descriptive Statistics for Executive Function Tasks and the SDQ*

|  | ***n*** | ***Mean*** | ***SD*** | **Min** | **Max** | **Skewness** | **Kurtosis** | **% Missing** |
| --- | --- | --- | --- | --- | --- | --- | --- | --- |
| *Inhibitory control (Flanker)* | 852 | 9.77 | 4.96 | 0 | 17 | -0.30 | -1.09 | 15% |
| *Cognitive flexibility (HTKS)* |  |  |  |  |  |  |  |  |
| HTKS – Part 1 | 963 | 16.77 | 4.50 | 0 | 20 | -2.41 | 5.53 | 4% |
| HTKS – Part 2 | 932 | 14.28 | 5.65 | 0 | 20 | -1.31 | 0.70 | 7% |
| HTKS – Part 3 | 867 | 9.83 | 6.99 | 0 | 20 | -0.07 | -1.40 | 13% |
| *Working memory (BAS)* |  |  |  |  |  |  |  |  |
| BAS – Part 1 | 990 | 1.73 | 0.61 | 0 | 2 | -2.07 | 2.84 | 1% |
| BAS – Part 2 | 989 | 0.97 | 0.81 | 0 | 2 | 0.06 | -1.49 | 1% |
| BAS – Part 3 | 985 | 0.19 | 0.43 | 0 | 2 | 2.21 | 4.23 | 2% |
| *Child Adjustment (SDQ)* |  |  |  |  |  |  |  |  |
| Externalizing problems | 969 | 5.66 | .105 | 0 | 17 | .581 | -.114 | 3% |
| Internalizing problems | 969 | 3.33 | .081 | 0 | 15 | 1.067 | 1.340 | 3% |

# **Table S4**

# *Information Criteria of the Uniform MIMIC-DIF Testing Procedure for Four Items of the EF Latent Variable, Model Comparisons, and DIF Effects (MLR estimation)*

| **Model** | **LL** | **Npar** | **AIC** | **BIC** | **aBIC** | **Uniform DIF effects**  **(HK vs. ENG)** | | | | **Uniform DIF effects**  **(MC vs. ENG)** | | | | **Uniform DIF effects**  **(MC vs. HK)** | | | |
| --- | --- | --- | --- | --- | --- | --- | --- | --- | --- | --- | --- | --- | --- | --- | --- | --- | --- |
|  |  |  |  |  |  | ***B*** | ***SE*** | ***p*** | ***b*** | ***B*** | ***SE*** | ***p*** | ***b*** | ***B*** | ***SE*** | ***p*** | ***b*** |
| Baseline | -13258.64 | 26 | 26569.28 | 26696.94 | 26614.36 | - | - | - | - | - | - | - | - | - | - | - | - |
| Uniform DIF (comparison group vs. reference group) | | | | | | | | | | | | | | | | | |
| BAS – Part 1 | -13246.91 | 28 | 26549.81 | 26687.29 | 26598.36 | .207 | .047 | .000 | .153 | .046 | .043 | .288 | .037 | -.161 | .042 | .000 | -.128 |
| BAS – Part 2 | -13250.37 | 28 | 26556.74 | 26694.22 | 26605.29 | -.277 | .056 | .000 | -.127 | -.089 | .053 | .095 | -.053 | .138 | .055 | .012 | .083 |
| HTKS – Part 2 | -13255.39 | 28 | 26566.77 | 26704.25 | 26615.32 | -.711 | .377 | .059 | -.057 | -.865 | .370 | .019 | -.074 | -.154 | .365 | .673 | -.013 |
| HTKS – Part 3 | -13247.60 | 28 | 26551.20 | 26688.68 | 26599.75 | 1.867 | .544 | .001 | .120 | 2.321 | .525 | .000 | .161 | .454 | .500 | .365 | .031 |

*Note*. Three-site region was re-coded as two dummy variables: reference group = 0, comparison group =1. LL = Log-likelihood value; Npar = number of parameters; AIC = Akaike's information criterion; BIC = Bayesian information criterion; aBIC = sample size-adjusted BIC. ENG = England; HK = Hong Kong; MC = mainland China.

# **Table S5**

# *Information Criteria of the Uniform MIMIC-DIF Testing Procedure for Nine Items of the Child Adjustment Latent Variables, Model Comparisons, and DIF Effects (MLR estimation)*

| **Model** | **LL** | **Npar** | **AIC** | **BIC** | **aBIC** | **Uniform DIF effects**  **(HK vs. ENG)** | | | | **Uniform DIF effects**  **(MC vs. ENG)** | | | | **Uniform DIF effects**  **(MC vs. HK)** | | | |
| --- | --- | --- | --- | --- | --- | --- | --- | --- | --- | --- | --- | --- | --- | --- | --- | --- | --- |
|  |  |  |  |  |  | ***B*** | ***SE*** | ***p*** | ***b*** | ***B*** | ***SE*** | ***p*** | ***b*** | ***B*** | ***SE*** | ***p*** | ***b*** |
| Baseline | -9682.41 | 50 | 19464.814 | 19708.936 | 19550.136 | - | - | - | - | - | - | - | - | - | - | - | - |
| Uniform DIF (comparison group vs. reference group) | | | | | | | | | | | | | | | | | |
| SDQ2 | -9658.47 | 52 | 19420.930 | 19674.817 | 19509.664 | -.278 | .042 | .000 | -.182 | -.164 | .037 | .000 | -.116 | .114 | .037 | .002 | .080 |
| SDQ5 | -9674.04 | 52 | 19452.072 | 19705.959 | 19540.807 | -.144 | .058 | .014 | -.096 | -.207 | .053 | .000 | -.149 | -.063 | .050 | .212 | -.045 |
| SDQ6 | -9669.08 | 52 | 19442.152 | 19696.039 | 19530.887 | -.125 | .043 | .004 | -.114 | -.192 | .038 | .000 | -.188 | -.067 | .035 | .053 | -.066 |
| SDQ7R | -9655.79 | 52 | 19415.571 | 19669.458 | 19504.305 | .144 | .044 | .001 | .115 | .308 | .043 | .000 | .264 | .163 | .044 | .000 | .140 |
| SDQ10 | -9674.58 | 52 | 19453.154 | 19707.041 | 19541.889 | .151 | .044 | .001 | .097 | .124 | .038 | .001 | .086 | -.027 | .037 | .468 | -.019 |
| SDQ15 | -9666.46 | 52 | 19436.922 | 19690.809 | 19525.657 | -.185 | .059 | .002 | -.122 | -.276 | .053 | .000 | -.195 | -.091 | .049 | .064 | -.064 |
| SDQ16 | -9668.62 | 52 | 19441.232 | 19695.119 | 19529.967 | .275 | .051 | .000 | .182 | .143 | .049 | .004 | .101 | -.133 | .048 | .005 | -.094 |
| SDQ24 | -9680.95 | 52 | 19465.893 | 19719.779 | 19554.627 | .073 | .044 | .098 | .057 | .025 | .042 | .550 | .021 | -.048 | -.039 | .214 | -.040 |
| SDQ25R | -9647.10 | 52 | 19398.202 | 19652.089 | 19486.937 | .353 | .042 | .000 | .242 | .266 | .041 | .000 | .196 | -.086 | .042 | .037 | -.064 |

*Note*. Three-site region was re-coded as two dummy variables: reference group = 0, comparison group =1. LL = Log-likelihood value; Npar = number of parameters; AIC = Akaike’s information criterion; BIC = Bayesian information criterion; aBIC = sample size-adjusted BIC. ENG = England; HK = Hong Kong; MC = mainland China.

# **Figure S1**

# *Histograms with Normal Curves for Executive Function Tasks*

S1a. Inhibitory control (Flanker): Full Sample


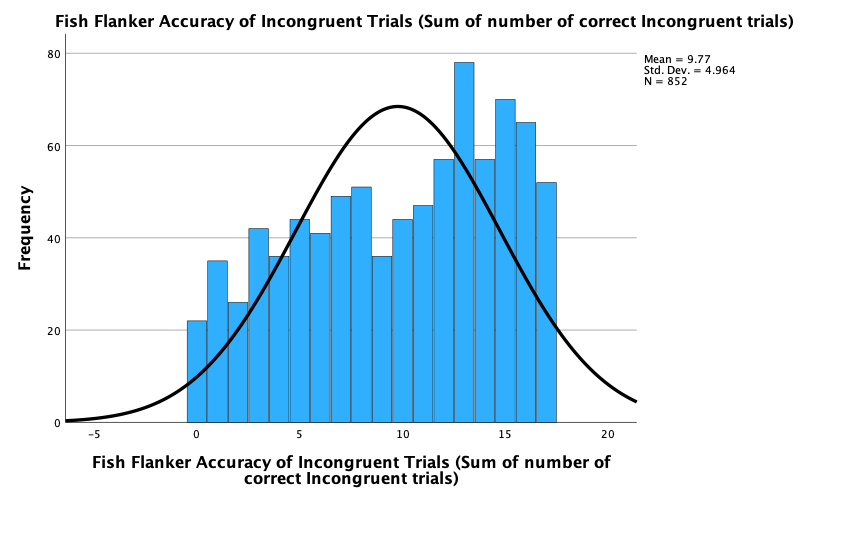


S1b. Inhibitory control (Flanker): England


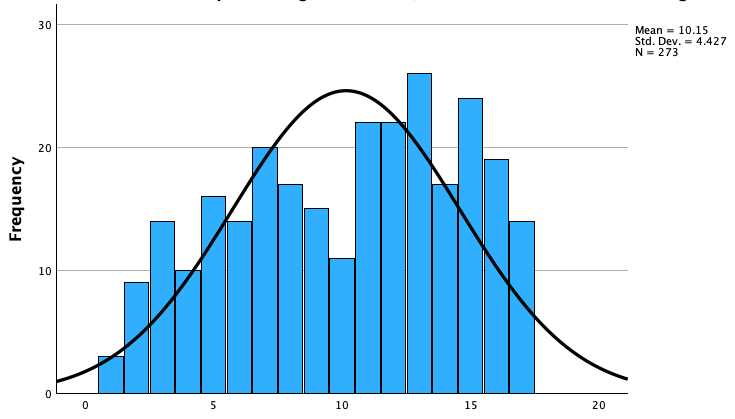


S1c. Inhibitory control (Flanker): Hong Kong


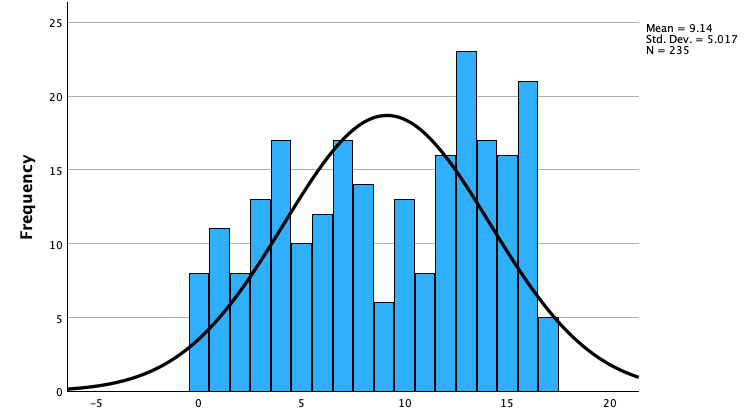


S1d. Inhibitory control (Flanker): Mainland China


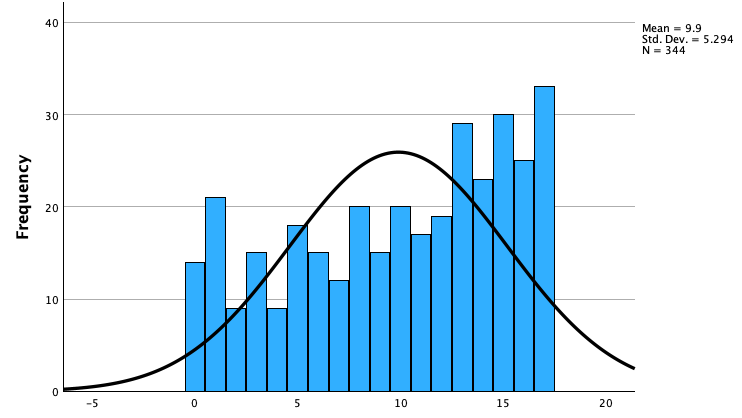


S1e. Cognitive flexibility (HTKS): Full sample


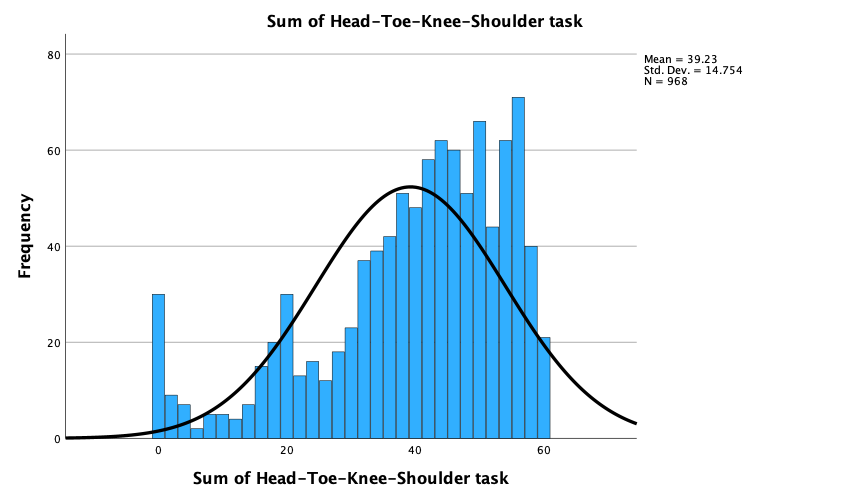


S1f. Cognitive flexibility (HTKS): England


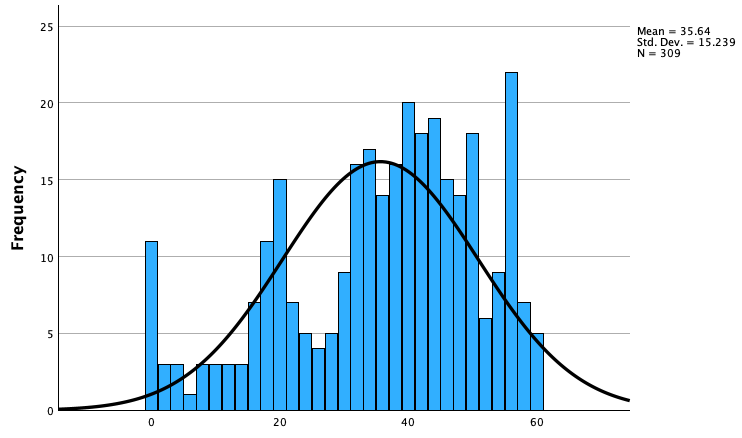


S1g. Cognitive flexibility (HTKS): Hong Kong


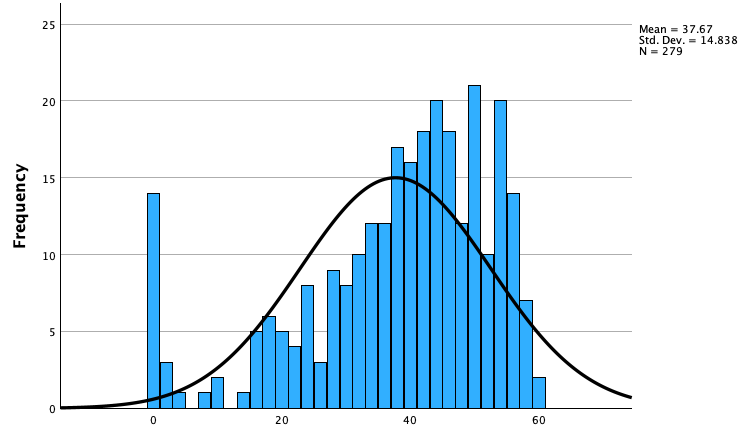


S1h. Cognitive flexibility (HTKS): Mainland China


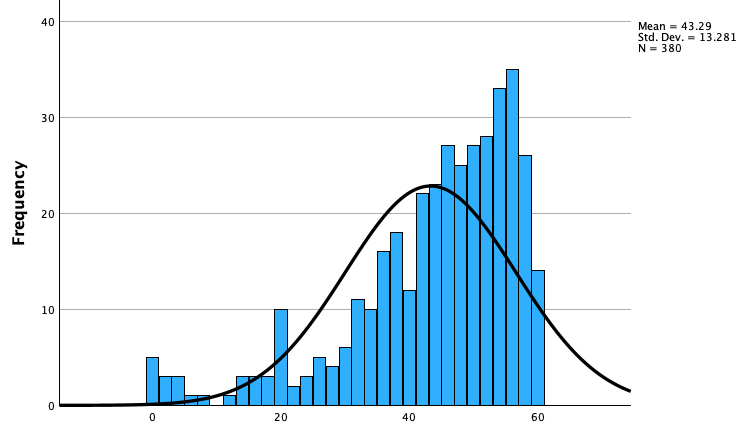


S1i. Working memory (BAS): Full sample


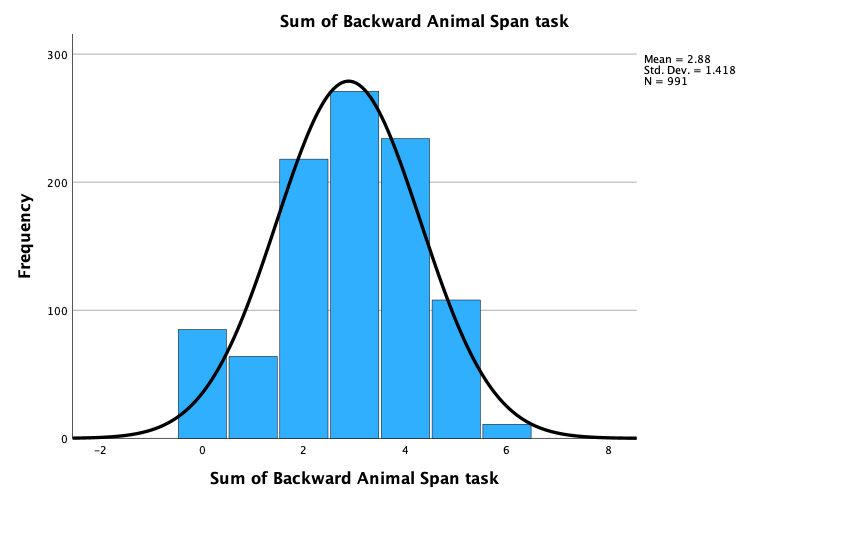


S1j. Working memory (BAS): England


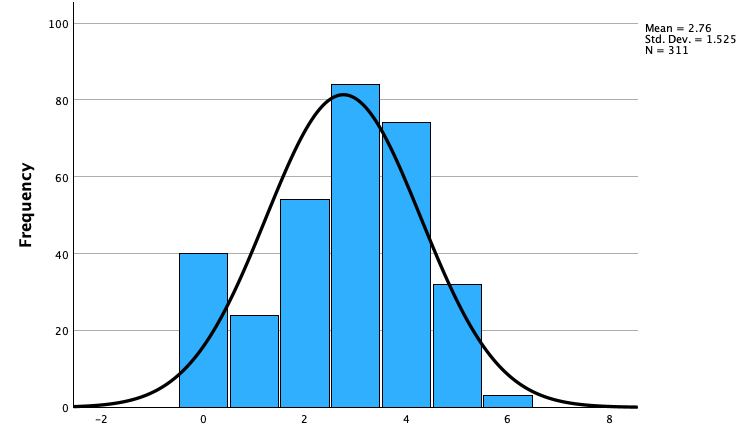


S1k. Working memory (BAS): Hong Kong


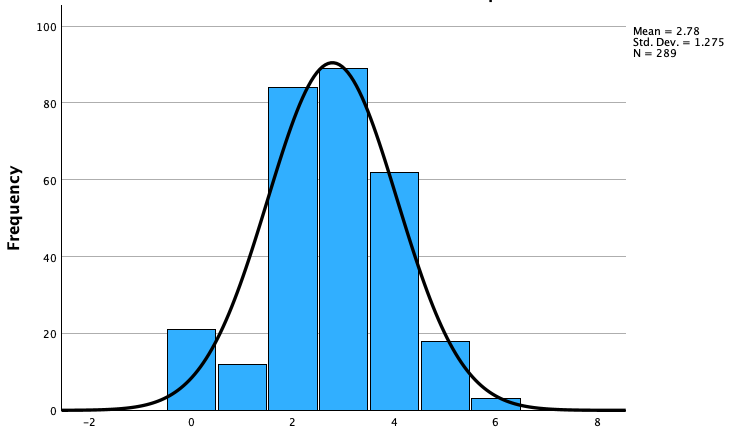


S1l. Working memory (BAS): Mainland China


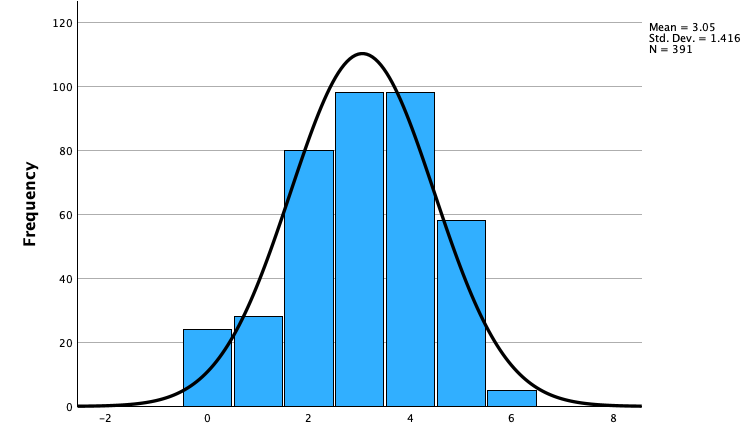


**Figure S2**

*Unstandardized Robust Maximum Likelihood Estimates for Paths between Executive Function and Adjustment Problems (With All Equality Constraints Placed)*
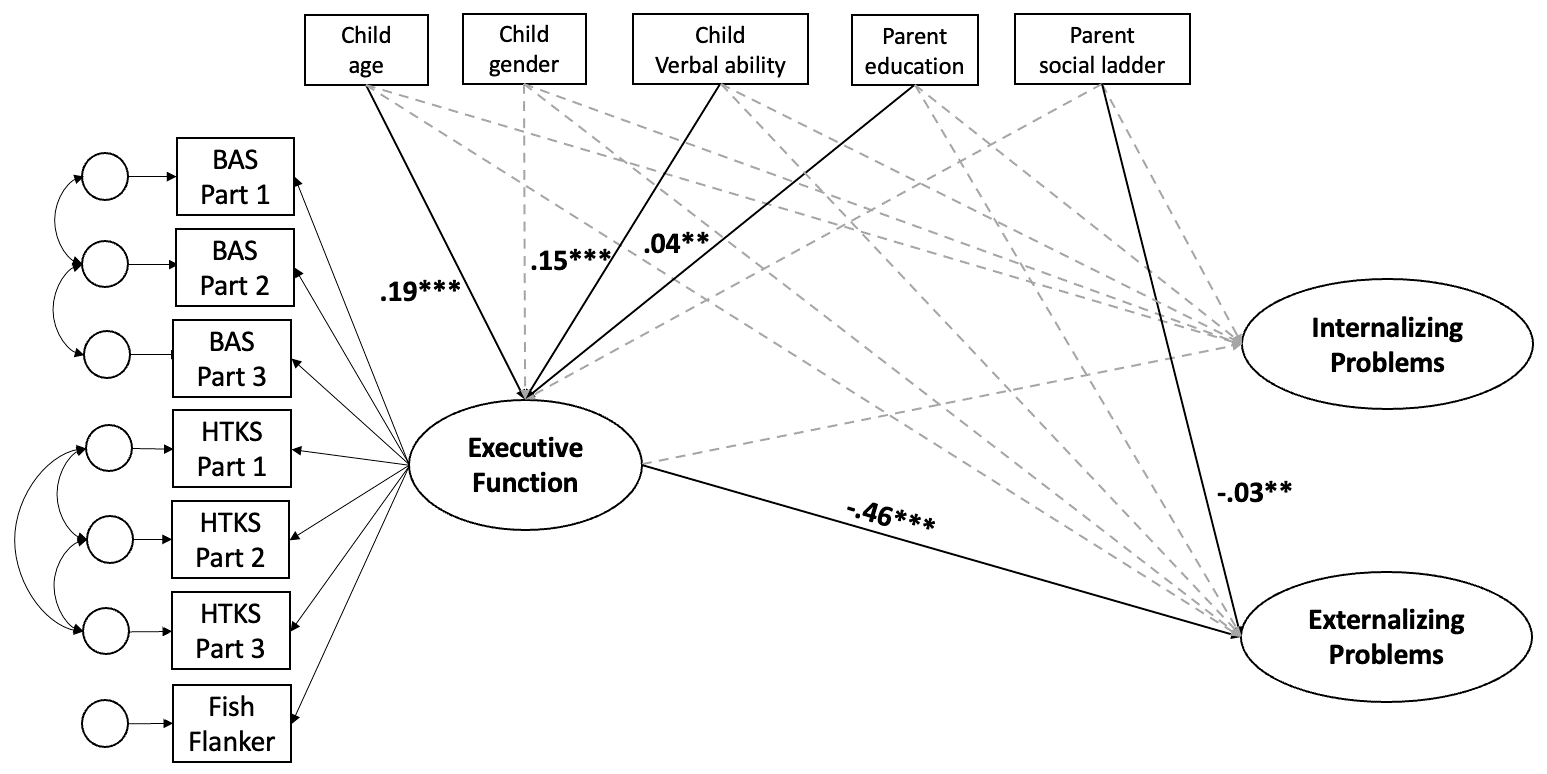


*Note.* Dash lines represent non-significant paths. Solid lines represent significant paths. ***p* < .01. ****p* < .001.

# **M*plus* Codes**

## 1. Configural, Metric, and Scalar Invariance Models of Executive Function

DATA:

FILE IS Anonymous dataset for mplus analysis.csv;

VARIABLE:

NAMES ARE MPLUSID REGION COHORT CGEN CAGE SEN PAGE SLAD PEDU HTT KST HTKST BASA BASB BASC FACCI SDQ1-SDQ7 SDQ7R SDQ8-SDQ11 SDQ11R SDQ12-SDQ14 SDQ14R SDQ15-SDQ21 SDQ21R SDQ22-SDQ25 SDQ25R ZVAC;

GROUPING IS REGION (1=HK 2=MC 3=UK);

IDVARIABLE IS MPLUSID;

USEVARIABLES ARE HTT KST HTKST BASA BASB BASC FACCI;

MISSING = ALL (-99);

!This statement indicates the performance of configural, metric, and scalar invariance models

ANALYSIS: ESTIMATOR = MLR;

MODEL = CONFIGURAL METRIC SCALAR;

MODEL:

EF BY BASA BASB BASC HTT KST HTKST FACCI;

!The following statements are made according to the output of modification indices

BASB WITH BASA;

BASC WITH BASB;

KST WITH HTT;

OUTPUT: STDYX RESIDUAL MODINDICES;

## 2. Partial Scalar Invariance Model of Executive Function

The partial scalar invariance model suggests not all item intercepts should be constrained to be equal across groups.

ANALYSIS: ESTIMATOR = MLR;

MODEL:

EF BY BASA BASB BASC HTT KST HTKST FACCI;

BASB WITH BASA;

BASC WITH BASB;

KST WITH HTT;

MODEL HK:

!Factor loadings are constrained to be equal across groups

EF BY BASB BASC HTT KST HTKST FACCI (a1-a6);

!Intercepts of BASA, BASB, KST, and HTKST were free across groups

[BASA* BASB* BASC* HTT* KST* HTKST* FACCI*] (b1-b7);

[EF@0];

MODEL MC:

!Factor loadings are constrained to be equal across groups

EF BY BASB BASC HTT KST HTKST FACCI (a1-a6);

!Intercepts of BASA, BASB, KST, and HTKST were free across groups

[BASA* BASB* BASC* HTT* KST* HTKST* FACCI*] (x1 x2 b3 b4 x5 x6 b7);

[EF*];

MODEL UK:

!Factor loadings are constrained to be equal across groups

EF BY BASB BASC HTT KST HTKST FACCI (a1-a6);

!Intercepts of BASA, BASB, KST, and HTKST were free across groups

[BASA* BASB* BASC* HTT* KST* HTKST* FACCI*] (y1 y2 b3 b4 y5 y6 b7);

[EF*];

OUTPUT: STDYX RESIDUAL MODINDICES;

SAVEDATA: SAVE = FSCORES;

FILE = 03_EF_1F_P.Scalar.csv;

MISSFLAG = 999;

## 3. Configural, Metric, and Scalar Invariance Models of Adjustment Problems

VARIABLE:

NAMES ARE MPLUSID REGION COHORT CGEN CAGE SEN PAGE SLAD PEDU HTT KST HTKST BASA BASB BASC FACCI SDQ1-SDQ7 SDQ7R SDQ8-SDQ11 SDQ11R SDQ12-SDQ14 SDQ14R SDQ15-SDQ21 SDQ21R SDQ22-SDQ25 SDQ25R

ZVAC;

GROUPING IS REGION (1=HK 2=MC 3=UK);

IDVARIABLE IS MPLUSID;

USEVARIABLES ARE SDQ5 SDQ7R SDQ2 SDQ10 SDQ15 SDQ21R SDQ25R

SDQ8 SDQ13 SDQ16 SDQ24 SDQ6 SDQ19;

MISSING = ALL (-99);

ANALYSIS: ESTIMATOR = MLR;

STARTS = 20;

MODEL = CONFIGURAL METRIC SCALAR;

MODEL:

EX BY SDQ5 SDQ7R SDQ2 SDQ10 SDQ15 SDQ21R SDQ25R;

IN BY SDQ8 SDQ13 SDQ16 SDQ24 SDQ6 SDQ19;

!The following statements are made according to the output of modification indices

SDQ10 WITH SDQ2;

SDQ24 WITH SDQ16;

SDQ25R WITH SDQ21R;

SDQ15 WITH SDQ7R;

SDQ15 WITH SDQ5;

SDQ25R WITH SDQ5;

OUTPUT: STDYX RESIDUAL MODINDICES;

## 4. Partial Metric Invariance Model of Adjustment Problems

The partial metric invariance model suggests not all factor loadings should be constrained to be equal across groups, and the item intercepts should be free.

ANALYSIS: ESTIMATOR = MLR;

STARTS = 20;

MODEL:

EX BY SDQ5

SDQ7R

SDQ2

SDQ10

SDQ15

SDQ21R

SDQ25R;

IN BY SDQ8

SDQ13

SDQ16

SDQ24

SDQ6

SDQ19;

SDQ10 WITH SDQ2;

SDQ24 WITH SDQ16;

SDQ25R WITH SDQ21R;

SDQ15 WITH SDQ7R;

SDQ15 WITH SDQ5;

SDQ25R WITH SDQ5;

MODEL HK:

!Free the factor loadings of SDQ7R, SDQ10, and SDQ24 across groups

EX BY

SDQ7R*

SDQ2 (13)

SDQ10*

SDQ15 (15)

SDQ21R (16)

SDQ25R (17);

IN BY

SDQ13 (19)

SDQ16 (20)

SDQ24*

SDQ6 (22)

SDQ19 (23);

!Free all the item intercepts across groups

[SDQ5* SDQ7R* SDQ2* SDQ10* SDQ15* SDQ21R* SDQ25R*];

[SDQ8* SDQ13* SDQ16* SDQ24* SDQ6* SDQ19*];

[EX@0 IN@0];

MODEL MC:

!Free the factor loadings of SDQ7R, SDQ10, and SDQ24 across groups

EX BY

SDQ7R*

SDQ2 (13)

SDQ10*

SDQ15 (15)

SDQ21R (16)

SDQ25R (17);

IN BY

SDQ13 (19)

SDQ16 (20)

SDQ24*

SDQ6 (22)

SDQ19 (23);

!Free all the item intercepts across groups

[SDQ5* SDQ7R* SDQ2* SDQ10* SDQ15* SDQ21R* SDQ25R*];

[SDQ8* SDQ13* SDQ16* SDQ24* SDQ6* SDQ19*];

[EX@0 IN@0];

MODEL UK:

!Free the factor loadings of SDQ7R, SDQ10, and SDQ24 across groups

EX BY

SDQ7R*

SDQ2 (13)

SDQ10*

SDQ15 (15)

SDQ21R (16)

SDQ25R (17);

IN BY

SDQ13 (19)

SDQ16 (20)

SDQ24*

SDQ6 (22)

SDQ19 (23);

!Free all the item intercepts across groups

[SDQ5* SDQ7R* SDQ2* SDQ10* SDQ15* SDQ21R* SDQ25R*];

[SDQ8* SDQ13* SDQ16* SDQ24* SDQ6* SDQ19*];

[EX@0 IN@0];

## 5. Partial Scalar Invariance Model of Adjustment Problems

Following the last partial metric invariance model, the partial scalar invariance model suggests the intercepts of items whose factor loadings are free should be free, in addition to other freely estimated item intercepts. Therefore, only a part of item intercepts would be constrained to be equal across groups.

ANALYSIS: ESTIMATOR = MLR;

STARTS = 20;

MODEL:

EX BY SDQ5

SDQ7R

SDQ2

SDQ10

SDQ15

SDQ21R

SDQ25R;

IN BY SDQ8

SDQ13

SDQ16

SDQ24

SDQ6

SDQ19;

SDQ10 WITH SDQ2;

SDQ24 WITH SDQ16;

SDQ25R WITH SDQ21R;

SDQ15 WITH SDQ7R;

SDQ15 WITH SDQ5;

SDQ25R WITH SDQ5;

MODEL HK:

EX BY

SDQ7R*

SDQ2 (13)

SDQ10*

SDQ15 (15)

SDQ21R (16)

SDQ25R (17);

IN BY

SDQ13 (19)

SDQ16 (20)

SDQ24*

SDQ6 (22)

SDQ19 (23);

!Constrain the following item intercepts to be equal across groups

[SDQ21R] (26);

[SDQ8] (28);

[SDQ13] (29);

[SDQ19] (33);

!Free the following item intercepts

[SDQ5* SDQ7R* SDQ10* SDQ15* SDQ25R* SDQ2*];

[SDQ16* SDQ24* SDQ6*];

[EX@0 IN@0];

MODEL MC:

EX BY

SDQ7R*

SDQ2 (13)

SDQ10*

SDQ15 (15)

SDQ21R (16)

SDQ25R (17);

IN BY

SDQ13 (19)

SDQ16 (20)

SDQ24*

SDQ6 (22)

SDQ19 (23);

!Constrain the following item intercepts to be equal across groups

[SDQ21R] (26);

[SDQ8] (28);

[SDQ13] (29);

[SDQ19] (33);

!Free the following item intercepts

[SDQ5* SDQ7R* SDQ10* SDQ15* SDQ25R* SDQ2*];

[SDQ16* SDQ24* SDQ6*];

[EX* IN*];

MODEL UK:

EX BY

SDQ7R*

SDQ2 (13)

SDQ10*

SDQ15 (15)

SDQ21R (16)

SDQ25R (17);

IN BY

SDQ13 (19)

SDQ16 (20)

SDQ24*

SDQ6 (22)

SDQ19 (23);

!Constrain the following item intercepts to be equal across groups

[SDQ21R] (26);

[SDQ8] (28);

[SDQ13] (29);

[SDQ19] (33);

!Free the following item intercepts

[SDQ5* SDQ7R* SDQ10* SDQ15* SDQ25R* SDQ2*];

[SDQ16* SDQ24* SDQ6*];

[EX* IN*];

OUTPUT: STDYX RESIDUAL MODINDICES;

SAVEDATA: SAVE = FSCORES;

FILE = 05a_SDQ_2F_HK_MC_UK_P.Scalar.csv;

MISSFLAG = 999;

## 6. The Uniform DIF Testing (MLR Estimation)

The following is an example of the DIF test on one item, with Hong Kong as reference group.

DATA:

FILE IS Anonymous dataset for mplus analysis_Dummy variables_hk.csv;

VARIABLE:

NAMES ARE MPLUSID REGION MC UK COHORT CGEN CAGE SEN PAGE SLAD PEDU HTT KST HTKST BASA BASB BASC FACCI SDQ1-SDQ7 SDQ7R

SDQ8-SDQ11 SDQ11R SDQ12-SDQ14 SDQ14R SDQ15-SDQ21 SDQ21R

SDQ22-SDQ25 SDQ25R ZVAC;

IDVARIABLE IS MPLUSID;

USEVARIABLES ARE MC UK HTT KST HTKST BASA BASB BASC FACCI;

MISSING = ALL (-99);

ANALYSIS: ESTIMATOR = MLR;

ALGORITHM = INTEGRATION;

H1ITEGRATIONS = 10000;

MODEL:

!Measurement model

EF BY BASA BASB BASC HTT KST HTKST FACCI;

BASB WITH BASA;

BASC WITH BASB;

KST WITH HTT;

!Structural (MIMIC) part

!Effects on the latent variable

EF ON MC UK;

!Item effects

BASA ON MC UK;

OUTPUT: STDYX; SAMPSTAT; CINTERVAL;

*Note*.

The baseline model is without the direct effects on the individual items (i.e., deleting the statement “BASA ON MC UK;”).

## 7. Multiple-Group Structural Equation Modeling without Constraints

For the multiple-group structural equation modeling based on the partial scalar models of executive function and adjustment problems.

DATA:

FILE IS Anonymous dataset for mplus analysis.csv;

VARIABLE:

NAMES ARE MPLUSID REGION COHORT CGEN CAGE SEN PAGE SLAD PEDU HTT KST HTKST BASA BASB BASC FACCI SDQ1-SDQ7 SDQ7R SDQ8-SDQ11 SDQ11R SDQ12-SDQ14 SDQ14R SDQ15-SDQ21 SDQ21R SDQ22-SDQ25 SDQ25R ZVAC;

GROUPING IS REGION (1=HK 2=MC 3=UK);

IDVARIABLE IS MPLUSID;

USEVARIABLES ARE CGEN CAGE SLAD PEDU HTT KST HTKST BASA BASB BASC FACCI SDQ5 SDQ7R SDQ2 SDQ10 SDQ15 SDQ21R SDQ25R SDQ8 SDQ13 SDQ16 SDQ24 SDQ6 SDQ19 ZVAC;

MISSING = ALL (-99);

ANALYSIS: ESTIMATOR = MLR;

MODEL:

!Below are the measurement models of executive function and adjustment models

EF BY BASA BASB BASC HTT KST HTKST FACCI;

BASB WITH BASA;

BASC WITH BASB;

KST WITH HTT;

EX BY SDQ5 SDQ7R SDQ2 SDQ10 SDQ15 SDQ21R SDQ25R;

IN BY SDQ8 SDQ13 SDQ16 SDQ24 SDQ6 SDQ19;

SDQ10 WITH SDQ2;

SDQ24 WITH SDQ16;

SDQ25R WITH SDQ21R;

SDQ15 WITH SDQ7R;

SDQ15 WITH SDQ5;

SDQ25R WITH SDQ5;

!Below is the structural model

EF ON CGEN CAGE SLAD PEDU ZVAC;

IN ON EF CGEN CAGE SLAD PEDU ZVAC;

EX ON EF CGEN CAGE SLAD PEDU ZVAC;

!The following WITH statements were made according to the output of modification indices

HTKST WITH KST;

HTKST WITH HTT;

!The following indicates the model of each group (aligned with the partial scalar

!invariance models)

MODEL HK:

EF BY BASB BASC HTT KST HTKST FACCI (a1-a6);

[BASA* BASB* BASC* HTT* KST* HTKST* FACCI*] (b1-b7);

[EF@0];

EX BY

SDQ7R*

SDQ2 (13)

SDQ10*

SDQ15 (15)

SDQ21R (16)

SDQ25R (17);

IN BY

SDQ13 (19)

SDQ16 (20)

SDQ24*

SDQ6 (22)

SDQ19 (23);

[SDQ21R] (26);

[SDQ8] (28);

[SDQ13] (29);

[SDQ19] (33);

[SDQ5* SDQ7R* SDQ10* SDQ15* SDQ25R* SDQ2*];

[SDQ16* SDQ24* SDQ6*];

[EX@0 IN@0];

MODEL MC:

EF BY BASB BASC HTT KST HTKST FACCI (a1-a6);

[BASA* BASB* BASC* HTT* KST* HTKST* FACCI*] (x1 x2 b3 b4 x5 x6 b7);

[EF*];

EX BY

SDQ7R*

SDQ2 (13)

SDQ10*

SDQ15 (15)

SDQ21R (16)

SDQ25R (17);

IN BY

SDQ13 (19)

SDQ16 (20)

SDQ24*

SDQ6 (22)

SDQ19 (23);

[SDQ21R] (26);

[SDQ8] (28);

[SDQ13] (29);

[SDQ19] (33);

[SDQ5* SDQ7R* SDQ10* SDQ15* SDQ25R* SDQ2*];

[SDQ16* SDQ24* SDQ6*];

[EX* IN*];

MODEL UK:

EF BY BASB BASC HTT KST HTKST FACCI (a1-a6);

[BASA* BASB* BASC* HTT* KST* HTKST* FACCI*] (y1 y2 b3 b4 y5 y6 b7);

[EF*];

EX BY

SDQ7R*

SDQ2 (13)

SDQ10*

SDQ15 (15)

SDQ21R (16)

SDQ25R (17);

IN BY

SDQ13 (19)

SDQ16 (20)

SDQ24*

SDQ6 (22)

SDQ19 (23);

[SDQ21R] (26);

[SDQ8] (28);

[SDQ13] (29);

[SDQ19] (33);

[SDQ5* SDQ7R* SDQ10* SDQ15* SDQ25R* SDQ2*];

[SDQ16* SDQ24* SDQ6*];

[EX* IN*];

OUTPUT: STDYX RESIDUAL MODINDICES;

## 8. Multiple-Group Structural Equation Modeling with Constraints

MODEL:

EF BY BASA BASB BASC HTT KST HTKST FACCI;

BASB WITH BASA;

BASC WITH BASB;

KST WITH HTT;

EX BY SDQ5 SDQ7R SDQ2 SDQ10 SDQ15 SDQ21R SDQ25R;

IN BY SDQ8 SDQ13 SDQ16 SDQ24 SDQ6 SDQ19;

SDQ10 WITH SDQ2;

SDQ24 WITH SDQ16;

SDQ25R WITH SDQ21R;

SDQ15 WITH SDQ7R;

SDQ15 WITH SDQ5;

SDQ25R WITH SDQ5;

EF ON CGEN CAGE SLAD PEDU ZVAC;

IN ON EF CGEN CAGE SLAD PEDU ZVAC;

EX ON EF CGEN CAGE SLAD PEDU ZVAC;

HTKST WITH KST;

HTKST WITH HTT;

MODEL HK:

EF BY BASB BASC HTT KST HTKST FACCI (a1-a6);

[BASA* BASB* BASC* HTT* KST* HTKST* FACCI*] (b1-b7);

[EF@0];

EX BY

SDQ7R*

SDQ2 (13)

SDQ10*

SDQ15 (15)

SDQ21R (16)

SDQ25R (17);

IN BY

SDQ13 (19)

SDQ16 (20)

SDQ24*

SDQ6 (22)

SDQ19 (23);

[SDQ21R] (26);

[SDQ8] (28);

[SDQ13] (29);

[SDQ19] (33);

[SDQ5* SDQ7R* SDQ10* SDQ15* SDQ25R* SDQ2*];

[SDQ16* SDQ24* SDQ6*];

[EX@0 IN@0];

!All paths are constrained to be equal across groups

EF ON CGEN CAGE SLAD PEDU ZVAC (m1-m5);

IN ON EF CGEN CAGE SLAD PEDU ZVAC (n1-n6);

EX ON EF CGEN CAGE SLAD PEDU ZVAC (n7-n12);

MODEL MC:

EF BY BASB BASC HTT KST HTKST FACCI (a1-a6);

[BASA* BASB* BASC* HTT* KST* HTKST* FACCI*] (x1 x2 b3 b4 x5 x6 b7);

[EF*];

EX BY

SDQ7R*

SDQ2 (13)

SDQ10*

SDQ15 (15)

SDQ21R (16)

SDQ25R (17);

IN BY

SDQ13 (19)

SDQ16 (20)

SDQ24*

SDQ6 (22)

SDQ19 (23);

[SDQ21R] (26);

[SDQ8] (28);

[SDQ13] (29);

[SDQ19] (33);

[SDQ5* SDQ7R* SDQ10* SDQ15* SDQ25R* SDQ2*];

[SDQ16* SDQ24* SDQ6*];

[EX* IN*];

!All paths are constrained to be equal across groups

EF ON CGEN CAGE SLAD PEDU ZVAC (m1-m5);

IN ON EF CGEN CAGE SLAD PEDU ZVAC (n1-n6);

EX ON EF CGEN CAGE SLAD PEDU ZVAC (n7-n12);

MODEL UK:

EF BY BASB BASC HTT KST HTKST FACCI (a1-a6);

[BASA* BASB* BASC* HTT* KST* HTKST* FACCI*] (y1 y2 b3 b4 y5 y6 b7);

[EF*];

EX BY

SDQ7R*

SDQ2 (13)

SDQ10*

SDQ15 (15)

SDQ21R (16)

SDQ25R (17);

IN BY

SDQ13 (19)

SDQ16 (20)

SDQ24*

SDQ6 (22)

SDQ19 (23);

[SDQ21R] (26);

[SDQ8] (28);

[SDQ13] (29);

[SDQ19] (33);

[SDQ5* SDQ7R* SDQ10* SDQ15* SDQ25R* SDQ2*];

[SDQ16* SDQ24* SDQ6*];

[EX* IN*];

!All paths are constrained to be equal across groups

EF ON CGEN CAGE SLAD PEDU ZVAC (m1-m5);

IN ON EF CGEN CAGE SLAD PEDU ZVAC (n1-n6);

EX ON EF CGEN CAGE SLAD PEDU ZVAC (n7-n12);

OUTPUT: STDYX RESIDUAL MODINDICES;
